# Palaeoecology of a billion‐year‐old non‐marine cyanobacterium from the Torridon Group and Nonesuch Formation

**DOI:** 10.1111/pala.12212

**Published:** 2015-11-09

**Authors:** Paul K. Strother, Charles H. Wellman

**Affiliations:** ^1^ Department of Earth and Environmental SciencesWeston Observatory of Boston CollegeWestonMA02493USA; ^2^ Department of Animal and Plant SciencesThe University of SheffieldSheffieldS10 2TNUK

**Keywords:** Precambrian, lacustrine, phosphogenesis, microcystins, Mesoproterozoic

## Abstract

A new chroococcalean cyanobacterium is described from approximately 1‐billion‐year‐old non‐marine deposits of the Torridonian Group of Scotland and the Nonesuch Formation of Michigan, USA. Individual cells of the new microfossil, *Eohalothece lacustrina* gen. et sp. nov., are associated with benthic microbial biofilms, but the majority of samples are recovered in palynological preparations in the form of large, apparently planktonic colonies, similar to extant species of *Microcystis*. In the Torridonian, *Eohalothece* is associated with phosphatic nodules, and we have developed a novel hypothesis linking *Eohalothece* to phosphate deposition in ancient freshwater settings. Extant cyanobacteria can be prolific producers of extracellular microcystins, which are non‐ribosomal polypeptide phosphatase inhibitors. Microcystins may have promoted the retention and concentration of sedimentary organic phosphate prior to mineralization of francolite and nodule formation. This has a further implication that the Torridonian lakes were nitrogen limited as the release of microcystins is enhanced under such conditions today. The abundance and wide distribution of *Eohalothece lacustrina* attests to the importance of cyanobacteria as oxygen‐producing photoautotrophs in lacustrine ecosystems at the time of the Mesoproterozoic–Neoproterozoic transition.

During an ongoing study of the palaeobiology of the Torridon Group of the north‐west Scottish Highlands (Strother *et al*. 2011) and the approximately coeval Nonesuch Formation of Michigan (USA) (Wellman and Strother 2015), we encountered a small, ellipsoidal organic‐walled microfossil which is common to both deposits. The new species, *Eohalothece lacustrina*, occurs in the Nonesuch Formation in palynological preparations as individual cells, small contiguous populations, but, primarily as large populations of varying gross morphology. It is also found in association with small to large sheets of granular/amorphous organic matter that appear to be highly degraded remains of microbial biofilms. In the Torridon Group, it occurs as dispersed palynomorphs in palynological preparations. But *Eohalothece* is also found in thin sections of phosphatic nodules and microbially induced sedimentary structures (miss) where, in some cases, populations of scattered individuals appear to be preserved *in situ*. This distribution encouraged us to explore in more detail the sedimentary dynamics and fabric of *in situ* preservation of reticulate miss in the Torridon Group, and we propose that raindrop impressions impacting a biofilm‐rich sediment were involved in producing the reticulate miss.

*Eohalothece* primarily occurs in colonies, some of which are strikingly similar to planktonic species of the extant cyanobacterium, *Microcystis* Kützing, –1849, including the widespread *M. aeruginosa* (van Gremberghe *et al*. 2011). So *Eohalothece* appears to have possessed a distinctly planktonic life mode. In the Torridonian, it is also found in phosphatized miss, but in this case, instead of large colonies, the individual cells of *Eohalothece* are scattered, seemingly randomly, within organic‐rich laminae. We have tried to find an explanation for this apparently disjunct ecology, that is populations of scattered solitary benthic individuals and distinctly colonial forms, although it is clear that taphonomy has played a role in presenting these seemingly different ecologies.

Recent and ongoing studies have pointed to a relationship between cyanobacteria and phosphogenesis during Precambrian time (Hubert 2005; She *et al*. 2014). Our observations of *Eohalothece* preserved in the Diabaig Formation show an apparent relationship between this fossil and diagenetic phosphate. The extant morphological analogue to *Eohalothece*,* Microcystis aeruginosa*, is a well‐known producer of microcystin, a phosphatase inhibitor. Microcystins have been proposed as contributing to Phanerozoic extinctions (Castle and Rodgers 2009), and detailed sedimentological and geochemical analyses have linked seasonal production of excess microcystins to mass deaths of animals in Pleistocene (Braun and Pfeiffer 2002) and Miocene (Koenigswald *et al*. 2004) lakes. We hypothesize that the growth and burial of these cyanobacteria in settings which lead to the excess production of microcystins may have promoted the trapping and retention of organic phosphorus into accumulating sediments. Given rapid burial and favourable redox conditions during early diagenesis, this may have promoted the formation of authigenic phosphate in the Diabaig shales.

## Geological setting

### The Torridonian

**Figure 1:**
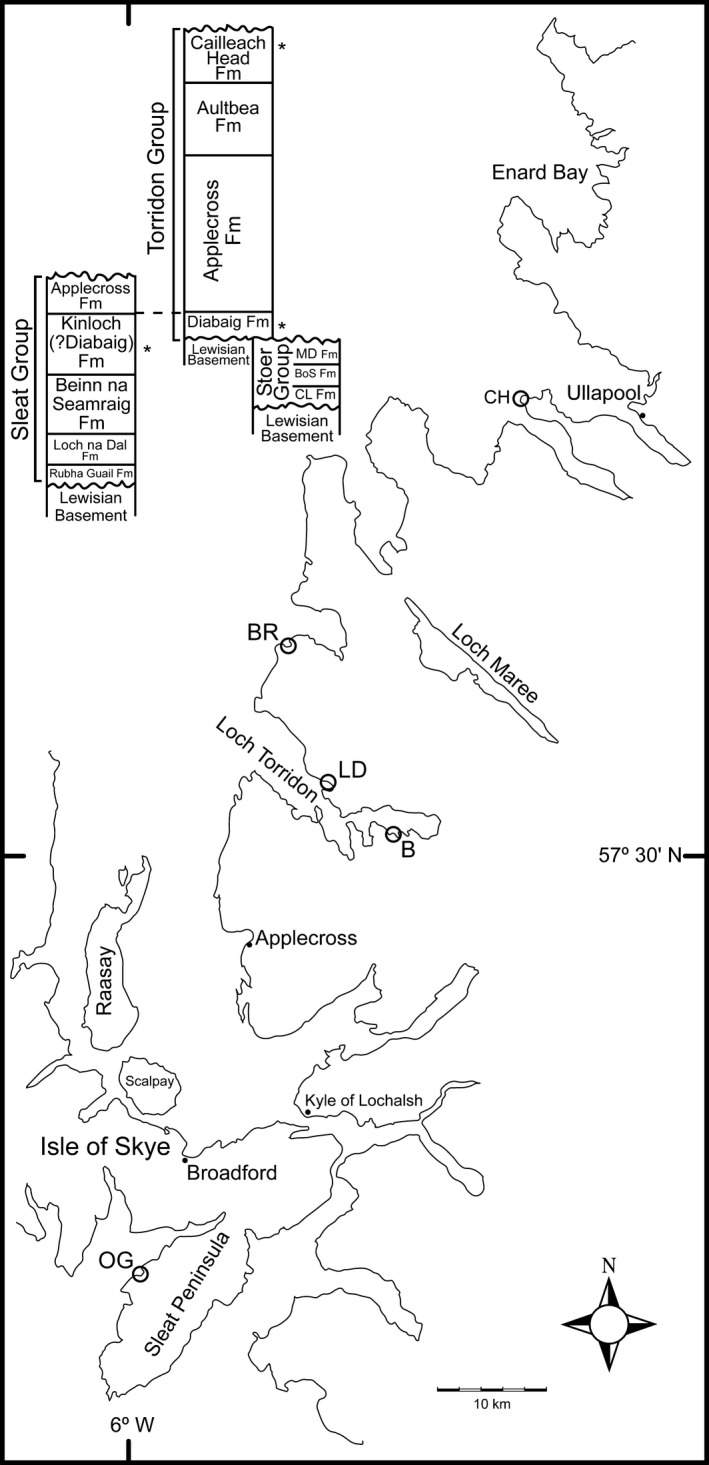
Location map and basic stratigraphic relations for the Torridon Group. *Abbreviations*: B, Balgy; BoS Fm, Bay of Stoer Formation; BR, Badachro River; CH, Cailleach Head; CL Fm, Clachtoll Formation; LD, Lower Diabaig; MD Fm, Meall Dearg Formation; OG, Ob Gauscavaig.

The Torridonian is an informal name for an extensive Proterozoic clastic sequence located in the north‐west Scottish Highlands (Fig. 1). More than 10 km of largely fluvial sediments deposited on an ancient Lewisian landscape are overlain unconformably by lower Palaeozoic carbonates (Peach *et al*. 1907). The Torridonian sequence is comprised of three lithologically related units: the Stoer, Sleat and Torridon groups (Fig. 1). The Stoer Group is a maximum of 2 km in thickness and consists predominantly of alluvial fan, fluvial, swamp/lake and aeolian deposits (Stewart 2002). It has a Pb–Pb age of 1199 ± 70 Ma (Turnbull *et al*. 1996), which has recently been confirmed by ^40^Ar/^39^Ar dating to 1177 ± 5 Ma by Parnell *et al*. (2011). The unconformably overlying Torridon Group is up to 7 km in thickness and consists of alluvial fan, fluvial and lake deposits, with rare bajada facies developed locally (Stewart 2002). Torridonian siltstones at the Diabaig stratotype have produced Rb–Sr whole rock dates of 994 ± 48 and 977 ± 39 Ma (Turnbull *et al*. 1996). The Sleat Group is up to 3.5 km in thickness and consists of fan‐delta, fluvial and lake deposits. It has not been dated, but the upper part (Kinloch Fm) appears on lithological and palynological (Strother *et al*. 2011) grounds to be equivalent to the Diabaig Fm in the lower part of the Torridon Group.

The entire Torridonian sequence is essentially non‐marine and consists primarily of terrestrial–fluviatile–lacustrine–aeolian red bed facies. It is dominated by conglomerates, red sandstones and red shales, with rare reduced dark shale/siltstone intercalations. The latter are usually thin and laterally discontinuous and probably represent overbank deposits or small ponds (Stewart 2002). Occasionally, they are thicker and laterally persistent and these are interpreted as the deposits of substantial lakes, a premise that is supported by studies of boron levels in illites (Stewart and Parker 1979). Soils are also developed at a few horizons (Retallack and Mindszenty 1994; Stewart 2002; Orihel *et al*. 2012), and raindrop impressions, discussed over 100 years ago (Peach *et al*. 1907), are still found in outcrops today (Fig. 4E; Strother *et al*. 2011). Torridon Group conglomerates show typical sedimentological characteristics of alluvial fan deposits, and Torridonian sandstones are predominantly fluvial with cross bedding and wet sediment deformation features, representing deposits of braided rivers (Selley 1965). The laminated red and grey shales/siltstones of the Torridon Group exhibit shallow water depositional features throughout, but these are especially acute in the basal unit, the Diabaig Formation (Peach *et al*. 1907; Strother *et al*. 2011). Both thinly bedded and nodular phosphate occurs in reduced shales from throughout the Torridonian sequence.

The grey shales and siltstones of the Torridon group have long been known to harbour microfossils which were first illustrated by Teall (*in* Peach *et al*. 1907). A controversial sighting of plant spores by Naumova and Pavlovsky (1961) was quickly rebutted by Downie (1961). Downie was well aware of the acritarchs found at Cailleach Head and at Diabaig which he noted in some more general papers on the nature and distribution of acritarchs (Downie 1973). The first well‐documented palynomorphs were recovered from the Stoer group by Cloud and Germs (1971). They found *Favososphaeridium* and some simple leiospheres, taxa which were later confirmed by Strother *et al*. (2011). Subsequent reports by Peat and Diver (1982) and Peat (1984) noted the presence of microfossils, but the taxonomic affinities of their specimens remained open. Zhang (1982) erected the taxon *Torridoniphycus lepidus* for what he considered to be a cyanobacterium (blue‐green alga) with a complex life cycle, from microfossils extracted from shales of the Aultbea Fm (Torridon Group). He documented the presence of smaller cells, some of which were referred to as spores, down to about 10 μm in diameter, but the bulk of specimens attributed to the genus, including the holotype, were significantly larger and more complex. Given the size range, complexity and morphological variety of the specimens attributed to *Torridoniphycus*, these would probably be interpreted to be of a eukaryotic nature today (Strother *et al*. 2011).

### The Nonesuch Formation

**Figure 2:**
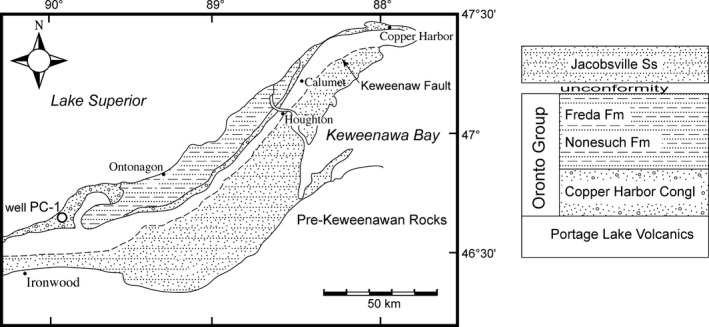
Location map and basic stratigraphic relations for the Nonesuch Formation. The holotype of *Eohalothece lacustrina* comes from Well PC‐1, which is noted on the map.

The Nonesuch Formation is a well‐known, native‐copper bearing formation of the Oronto Group found in the Mid‐continent Rift System in the Keweenaw Peninsula in the Upper Peninsula of Michigan, USA. Chocolate‐to‐dark grey‐coloured shales and siltstones preserving millimetre‐scale laminations are sandwiched conformably between the underlying Copper Harbor Conglomerate and the overlying red Freda Sandstone. The unit is somewhat limited in distribution; it is found in outcrop on the Wisconsin side of the southern shore of Lake Superior and it extends about 200 km east onto the Keweenaw Peninsula. The Nonesuch Formation is less than 200 m thick at the middle of its distribution in Gogebic County, Michigan. The cores and outcrop used in this report are from this region (Fig. 2).

The Nonesuch Formation has a direct Re–Os age of 1078 ± 24 Ma (Cumming *et al*. 2013) which is further constrained by a maximum age of 1087.2 ± 1.6 Ma from a basalt flow within the conformably underlying Copper Harbor Conglomerate (Davis and Paces 1990) and a minimum Rb–Sr date of 1047 ± 35 Ma from crosscutting calcite veins (Ruiz *et al*. 1984). The unit has long been considered to be an ancient lake, based on numerous lines of regional, sedimentological and mineralogical evidence (Elmore *et al*. 1989; Suszek 1997; Pedentchouk *et al*. 2004). Some early organic geochemical studies were consistent with a non‐marine source for the organic fraction of the Nonesuch Formation (Meinschein *et al*. 1964; Barghoorn *et al*. 1965). But others, focusing on the requirement for sulphur (Hieshima and Pratt 1991) and biomarkers (Pratt *et al*. 1991), concluded that the Nonesuch Formation must have been flushed with marine waters. Cumming *et al*. (2013) addressed these findings specifically and pointed out that S/C ratios can be supplemented by sulphur that is released from organic matter and that 24‐n‐propylcholestane (the eukaryotic biomarker noted by Pratt *et al*. (1991)) is not exclusively marine in distribution. Additional geochemical evidence of radiogenic Os unambiguously indicates a non‐marine Os source to the deposit (Cumming *et al*. 2013). Given that the palaeogeographical position of this part of the Midcontinent Rift System is thought to have been 800 km from the ocean (Cumming *et al*. 2013), and the undeniable sedimentological and regional geological settings, it is difficult to not to conclude that the dark laminated siltstone/shale of the Nonesuch represents an ancient lake deposit.

Microfossils recovered previously from the Nonesuch Formation have been somewhat disappointing. In a study emphasizing the organic geochemistry and biological provenance of organic matter from the base of the formation, Barghoorn *et al*. (1965) illustrated several spheroidal structures and some ‘plant’ tissues, but none of this material appears convincingly of plant origin today. Palynological preparations of the Nonesuch shales often release large sheets of organic matter which can be distorted from mineral grain impactions to yield pseudocellular patterns. Moore *et al*. (1969) described what they interpreted to be actinomycete fungi from the Nonesuch, but these too do not look convincing upon re‐examination. Strother (1994) illustrated several sphaeromorph acritarchs including *Trachysphaeridium* sp. and noted that filamentous tubes (sheaths), similar to *Siphonophycus*, were also present in the Nonesuch Formation. But the totality of this prior body of work left the distinct impression that the microfossil assemblage of the Nonesuch consisted of little but ‘sticks and balls’.

It appears that previous palaeontological examinations focused on samples collected in the White Pine mine or quite nearby. Contrariwise to all previous microbiological studies, a series of samples from wells PC‐1 and WPB‐4 collected in 2009 contain rich assemblages of well‐preserved palynomorphs (Wellman and Strother, 2015). Excellent preservation occurs in the upper portions of each core; sample NON09‐3, the source of the holotype of *Eohalothece*, is located 19 m from the upper (gradual) contact with the overlying Freda Sandstone. In that same core, the lowermost sample (NON09‐18) that contains a well‐preserved acritarch assemblage occurs 31 m above the contact with the underlying Copper Harbor Conglomerate. This is well above the basal unit that is mined for copper at White Pine, which extends only 6 or 7 m above the basal contact with the Copper Harbor Conglomerate. The simple conclusion, which is reinforced by an earlier organic geochemical study (Ho *et al*. 1990), is that the mineralization that has occurred at the base of the formation has degraded the bedded organic component resulting in poor microfossil recovery from this part of the section.

## Material and methods

Field samples were collected from throughout the Torridon Group (Strother *et al*. 2011), and core samples were collected from a number of cores (Wellman and Strother 2015) from the Nonesuch Formation. The samples were processed using standard HCl–HF palynological acid maceration techniques, followed by heavy mineral separation using ZnCl_2_, and sieving using a 10‐μm mesh. No oxidation techniques were utilized. Slides of the palynological preparations were examined using light microscopy. Thin sections, prepared from hand specimens of phosphate nodules and miss from the Torridon group, were examined using light microscopy. All materials are housed in the collections of the Centre for Palynology of the University of Sheffield. Slide locations are indicated using England Finder (EF) coordinates.

## Systematic palaeontology

#### Domain BACTERIA Woese *et al*., 1990 Kingdom EUBACTERIA Woese and Fox, 1977 Phylum CYANOBACTERIA Stanier *et al*., 1978 Class COCCOGONEAE Thuret, 1875 Order CHROOCOCCALES Wettstein, 1924 Family CHROOCOCCACEAE Nägeli, 1848 Genus EOHALOTHECE nov.

###### Type species

*Eohalothece lacustrina* sp. nov.

###### Derivation of name

From the Latin *eo* (dawn) and the extant genus, *Halothece*. The genus name is considered to be feminine.

###### Diagnosis

Organic‐walled microfossil, elliptical to subelliptical to rounded fusiform with slightly tapered ends; walls single‐layered but not particularly distinct, without folds; surface smooth to granular; cells small, solitary, in colonies of loosely clumped cells, or in large colonies that are globular to clathrate in form.

###### Remarks

This genus differs from *Eosynechococcus* Hofmann, 1976, in that the lateral sides are not always parallel to each other, but instead, they often taper very slightly towards the cell apices, resulting in a rounded fusiform shape. The cells of *Eosynechococcus thuleensis* Strother *et al*., 1983, which is only found as petrifactions in cherts, tend to line up end‐to‐end in chains of up to four cells as noted by Strother *et al*. (1983). This division pattern is notable given the name of the extant genus, *Synechococcus* Nägeli, 1848, as it describes exactly the topology of cells of the fossil genus, *Eosynechococcus* Hofmann, 1976, which tend to remain attached end‐to‐end, like sausage links. The extant genus *Synechococcus* is described by Desikachary (1959, p. 143) as occurring in ‘… colonies of 2, rarely in fours…’? Cells of *Eohalothece* tend to form much larger clusters of cells which are much more irregular in their arrangement, typically forming three‐dimensional colonies. It differs from *Paleophycus* Schopf, 1968, in its colonial habit and the predominantly elliptical to rounded fusiform shape. *Brachypleganon* Lo, 1980, is a small cylindrical form sometimes with open ends or rounded ends. The sides are parallel to each other, never tapering as in the rounded fusiform shape seen here in *Eohalothece*. *Eoaphanocapsa* Nyberg and Schopf, 1984, consists of colonies surrounded by a common envelope. Colonies of *Eohalothece* never possess a surrounding envelope or obvious enclosing sheath; the colonial cells are always free. The individual cells of *Eoaphanocapsa* are larger and more ovoid in shape than those of the new genus. *Gloeotheceopsis* Zhang, 1988, occurs as small colonies enclosed in a common sheath. All of the above taxa are described from cherts, and none have been found associated with lacustrine deposits. In addition, none of these taxa have been described previously from palynological preparations.

#### *Eohalothece lacustrina* sp. nov. Figures 3, 7, 8

###### Derivation of name

From the Latin *lacustrinus* (of lakes).

###### Holotype

**Figure 3:**
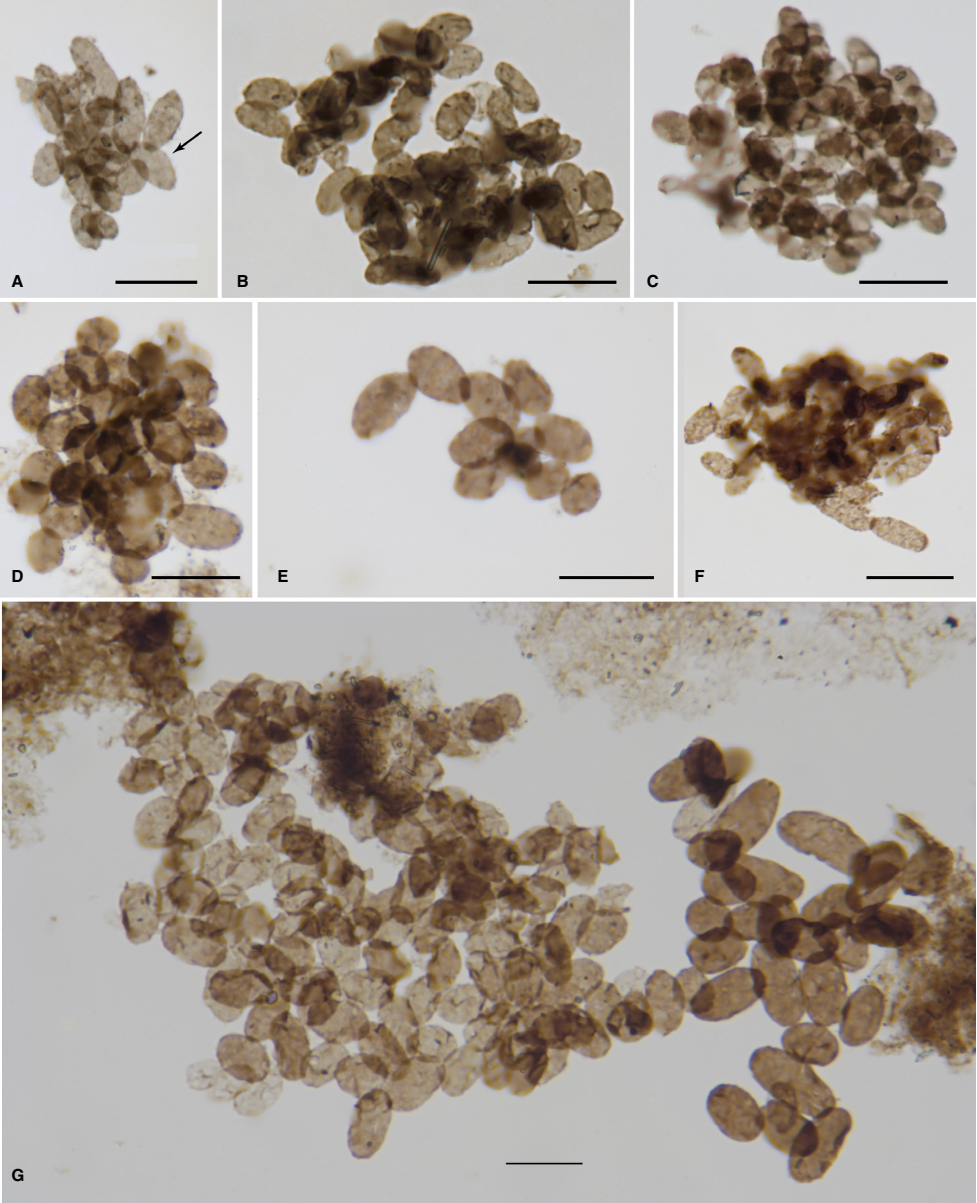
*Eohalothece lacustrina* gen. et sp. nov. populations from the Nonesuch Formation in well PC‐1. A, *Eohalothece lacustrina* holotype (arrow), slide NON09‐3B, EF loc. E52. B, *E. lacustrina* slide N0N09‐3B, paratypes. C, *E. lacustrina* slide N0N09‐3B, paratypes. D, *E. lacustrina* slide N0N09‐3B, paratypes. E, *E. lacustrina*, slide NON09‐3B, paratypes. F, *E. lacustrina*, slide NON09‐3 B, paratypes. G, *E. lacustrina* slide N0N09‐3B, paratypes. All scale bars represent 10 μm. Colour online.

The population illustrated in Figure 3A contains the holotype, which is marked on the figure by an arrow. Slide NON09‐3B, EF loc. U52. This slide is curated in the collections of the Centre for Palynology of the University of Sheffield, UK.

###### Type locality

The type material comes from core PC‐1, drilled in Gogebic Co, Michigan (46° 40.57′N 89° 58.97′E). At this locality, the Nonesuch Formation has a thickness of 201 m. Sample NON09‐3B was located near the top of the section, 19.8 m below the contact with the overlying Freda Sandstone.

###### Material

This new species has been observed in the following cores of the Nonesuch Formation. PC‐1, PI‐2, WPB‐2, WPB‐5, WPB‐7, and WPB‐8. It occurs in samples of Nonesuch Formation collected from outcrop below Manido Falls on a dry branch of the Presque Isle River in the Upper Peninsula Michigan (46° 42.498′N 89°58.325′W). In the Torridonian, additional specimens have been observed in phosphatic nodules from the Diabaig Formation at its type locality (Lower Diabaig, Scotland) and from macerated shale samples from the Kinloch Formation (sample TOR08‐11 at Ob Gauscavaig near Toskavaig on Skye, Scotland) and from the Cailleach Head Formation at Cailleach Head, Scotland (sample TOR08‐45).

###### Diagnosis

A species of *Eohalothece* with cells measuring up to 10 μm in length but typically 5–7 μm in length and 3–4 μm in width, walls of medium density and colour.

###### Description

The mean length of cells from six different sample populations ranged from 5.5 to 9.5 μm. The mean width of those same populations ranged from 3.3 to 5.6 μm. The ratio of width to length (ellipticity) of the same populations ranged from 0.58 to 0.72.

###### Remarks

This species most typically occurs in clusters of what appear to be genetically related populations of cells, and several populations were measured for characterization and comparison. The results of these descriptive statistics are presented in Table 1. We compared ellipticity with length to get a sense of elongation of these cells during growth. We used Excel to fit lines to the data and found that in all cases the highest r^2^ values were obtained using a logarithmic function. Given that these r^2^ values ranged from 0.65 to 0.90 (p < 0.001), this data lends support to the inference that *Eohalothece* is largely preserved as populations of cells that originally were together in a logarithmic growth phase. Three additional examples of populations with their length/ellipticity plots can be found in the online Supporting Information, Figure S1.

**Table 1:** Descriptive statistical comparisons between separate colonies of *E. lacustrina*

Image	Sample	N	Width (μm)	Length (μm)	W/L (μm)	Skewness	Logistic fit (r^2^)^a^
Figure 3A	NON09‐3	20	3.5	6.4	0.58	0.63	0.90
Figure 3E	NON09‐3	11	4.3	6.4	0.69	0.34	0.76
Figure 3G	NON09‐3	30	5.6	9.5	0.61	0.84	0.83
Figure S1A	NON09‐3	30	4.9	7.0	0.71	0.22	0.71
Figure S1B	NON09‐24	29	3.3	5.5	0.62	−0.18	0.69
Figure S1C	NON09‐24	40	4.4	6.3	0.72	0.90	0.65
Figure 7A	CW1C	52	3.7	6.0	0.63	0.14	0.26

a p < 0.001 in all cases.

The shape of individual cells of *Eohalothece* provides specific clues as to the probable cyanobacterial provenance of the taxon and, in addition, help distinguish populations of these small cells from previously described taxa such as *Paleophycus* and *Eosynechococcus*. Whether viewed in thin section (Fig. 7A–D) or extracted via acid maceration (Fig. 7E), individual cells always appear to be flattened without signs of folds or wrinkles. A similar condition occurs in the tubular sheaths of dispersed *Siphonophycus*,* Eomycetopsis* and related fossil cyanobacteria, which tend not to fold and wrinkle as they are compressed during compaction and subsequent lithification. For example, Figure 7H, which contains a biofilm with both *Siphonophycus* sp. and *E. lacustrina*, neither of which shows folding or other compressional artefacts. This appearance without folds, crests or thickenings in the compressed wall is different from many sphaeromorph acritarchs, such as *Leiosphaeridia crassa*, which retain large arcuate folds when their walls are taphonomically compressed, or *L. ternata* (Fig. 7A, F–G) which splits radially when compressed. These compression artefacts are due, in part, to the rigidity of thicker cell walls or cyst walls that are typically associated with eukaryotes. With cyanobacteria, the original, polysaccharide‐rich sheath and thinner, underlying cell walls typically exhibits plastic, not rigid, deformational behaviour during taphonomic compression. Living cyanobacteria of the *Euhalothece* group collected from the highly alkaline Lake Magadi (Kenya) have also been found to preserve under experimental fossilization conditions in the laboratory (Samylina and Gerasimenko 2011). The specific conditions of such experiments do not stand as models of the chemistry of ancient Torridonian lakes, but they do demonstrate that cyanobacteria of the *Euhalothece* Group are capable of surviving fossilization. These observations lead us to infer that the cell boundary in *Eohalothece* probably represents the remains either of the sheath or a thin cell wall, which surrounded the original cyanobacterial cell, rather than a more rigid, eukaryotic‐type wall.

The shape of the outline of *Eohalothece* is interesting as well. The cells are largely subelliptical in outline, but in some cases the ends appear somewhat more tapered, not so much as to be acuminate, but enough to distinguish them from typical rod‐shaped eubacteria. This can be seen clearly in the individual specimens illustrated at 1500× in Fig. 7B–E. This somewhat tapered, or rounded fusiform shape can also occur in extant *Aphanothece* and in the extant *Halothece–Euhalothece* complex of halophytic cyanobacteria (Garcia‐Pichel *et al*. 1998). Thus, gross cell shape adds to the rationale for classifying this fossil with the chroococcalean cyanobacteria.

## Discussion

### On the nature of reticulate miss in the Torridonian

In the shales at Lower Diabaig, the stratotype for the Diabaig Formation, many individual bedding planes display a distinctive reticulate miss that can be found throughout a nearly 200 m section. These have been described previously as miss by Prave (2002), Callow *et al*. (2011) and Strother *et al*. (2011), but in fact these patterned surfaces have a long history of varying interpretations, extending back to Hinxman (in Peach *et al*. 1907). Upfold (2009) considered reticulated patterns found in carbonates in the Stoer Group, which underlies the Torridon Group, to be analogues of tufted stromatolites occurring in Laguna Mormona (now Laguna Figueroa), Baja California, Mexico. His proposed that the preservation of the raised sedimentary pattern at Stoer was due to early cementation by carbonate, which was then responsible for preserving relief prior to compaction. However, the miss at Diabaig is not cemented by carbonate, so his carbonate cementation model does not stand as generally applicable for the origin of reticulate miss. Instead, the patterning is due to the ridges of silt and fine sand which remain proud of the postcompactional surface, with a combination of matrix, silica and phosphate functioning to cement the sediment.

**Figure 4:**
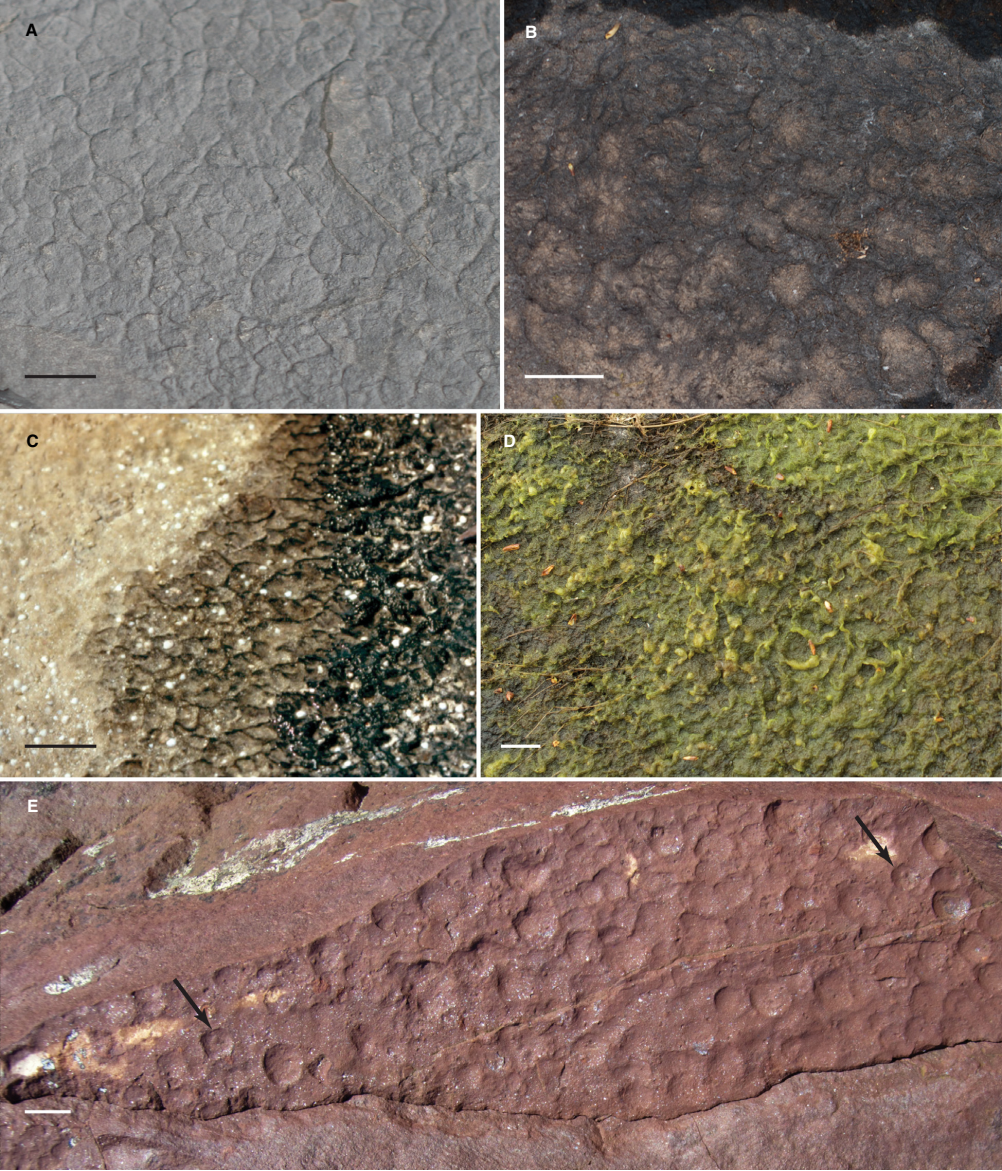
Comparative ancient miss, living microbial, extant and ancient raindrop impressions. A, reticulate miss surface from the Diabaig Formation at its type locality, Lower Diabaig, Scotland. B, dried extant microbial mat demonstrating reticulate texture formed by raindrops, Badachro River, Scotland. C, living tufted cyanobacterial (*Lyngbya*) mat at Hamlin Pool, Shark Bay, Western Australia, showing its transition to reticulate form and then to unoccupied sandy substrate. D, living algal mat demonstrating the reticulate pattern which occurs after a rain storm, Badachro River, Scotland; fully desiccated version of this mat, less than a metre distant, is seen in B. E, ancient raindrop impressions in the Stoer Group at a locality on the south shore of Enard Bay, Scotland, detail is fine enough that several impressions retain a central uplift formed during soft‐sediment impact (arrows), notice that overlapping impressions can form a reticulate pattern, similar to that seen in ancient miss in A. All scale bars represent 1 cm, except that in C which represents 2 cm. Colour online.

The reticulate pattern forms a network of cm‐scale ridges (Fig. 4A) which certainly do mimic the living tufted *Lyngbya* (Browne *et al*. 2000) mats seen today along the shoreline at Hamelin Pool at Shark Bay, Western Australia (Fig. 4C). The rather striking similarity between living tufted microbial mats and the reticulate miss at Diabaig turn out to be somewhat illusory, in part because the comparison is between the appearance of a surficial layer of (living) microbial mat and that of a lithified sediment. As noted by Upfold (2009), it is the early cementation by carbonate that preserves relief the primary (living) microbial mat. Without early cementation (prior to sedimentary compaction), the relief seen in the primary surface would be compacted and flattened. In modern day stromatolites, there is a similar requirement for penecontemporaneous cementation by carbonate (Reid *et al*. 2000) in order to preserve the synoptic profiles that characterize the classic, convex‐upward, club‐shaped stromatolites seen in the rock record.

**Figure 5:**
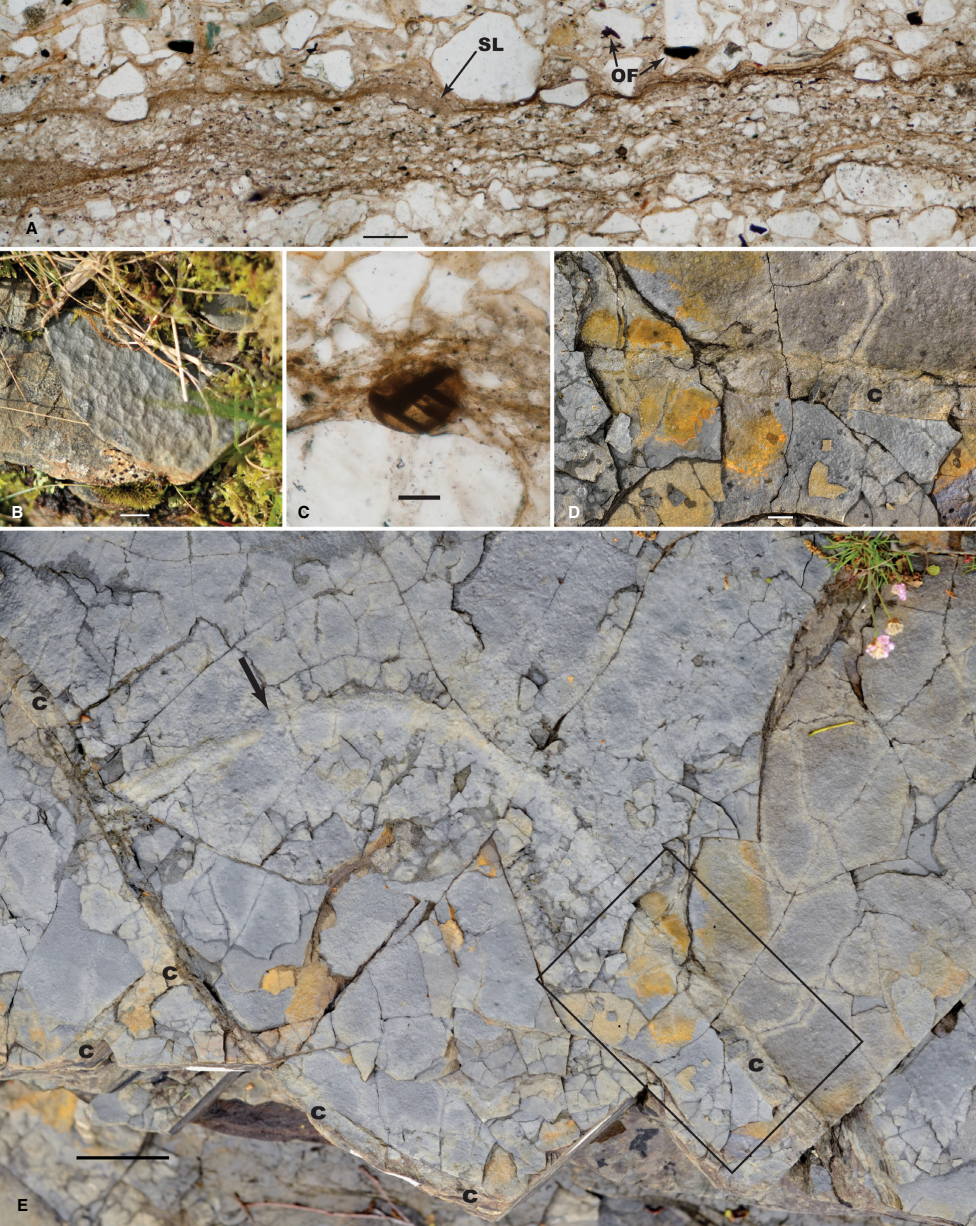
Field and thin‐section comparison of reticulate miss at Loch Diabaig, Scotland. A, petrographic thin section of the reticulate miss in transmitted plain white light showing the original surface layer (SL) of the primary mat and scattered organic fragments (OF); notice the lack of aligned or *in situ* filamentous (sheath) remains, sample TOR11‐113. B, outcrop photograph of the reticulate miss sectioned in A, sample TOR11‐113, Camas a’ Chlársair (Balgy), Scotland. C, organic‐walled microfossil (*Leiosphaeridia* cf. *L. crassa*) preserved in the zone underlying the surface layer shown in A. D, close‐up of miss‐covered mud crack (c) indicated by the boxed outline in E, demonstrating that the infilled mud crack clearly crosscuts the reticulate miss surface, Diabaig type locality at Lower Diabaig, Scotland. E, polygonal mud cracks both crosscut (Fig. 5D) and are covered by (arrow) reticulate miss indicating that these sediments were subaerially exposed and dried out as cracks (c) opened up and were subsequently infilled with sand, type section of the Diabaig Formation, Lower Diabaig, Scotland. Scale bars represent 100 μm (A); *c*. 1 cm (B); 25 μm (C); 0.8 cm (D); and 5 cm (E). Colour online.

**Figure 6:**
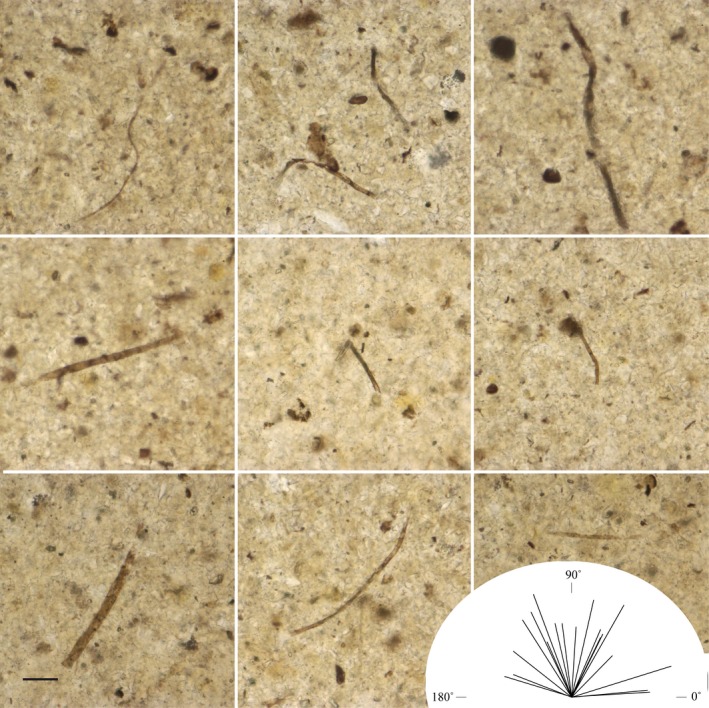
Composite of nine images of filament (sheath) fragments found in a bedding‐parallel thin section of a Diabaig phosphate nodule with a rose diagram showing that the filaments in this section do not display a preferred alignment. The length of the lines in the rose diagram is proportional to filament fragment length, n = 18. Although sheaths of what are likely cyanobacteria are scattered through the section, they appear to be allochthonous and are not evidence of an *in situ* mat‐building community. Sample TOR08‐26, type section of the Diabaig Formation, Lower Diabaig, Scotland. Scale bar represents 10 μm. Colour online.

When the Diabaig miss is examined in transverse thin section (Fig. 5A), there is no *in situ* preservation of filamentous cyanobacteria as one might expect if the tufted habit of the living analogue were directly responsible for the resultant sedimentary fabric. Figure 5A shows the *in situ* remains of a reticulate miss from a partially phosphatized sample (Fig. 5B) of the Diabaig Formation at Camas a’ Chlársair on the south shore of Loch Torridon. The lamination is due to interbedded coarser and finer layers, both of which are poorly sorted and both of which include clays and silt to fine sand clasts which can be quite angular. The finer layers, cemented with either apatite or silica, show organic matter which may be preserved either in the form of a condensed surface layer (SL) (Fig. 5A) or as structural organic fragments (OF). Only occasionally are recognizable cellular remains found in transverse thin sections (Fig. 5C). We have examined many individual laminae and have yet to find a single example in which any filamentous microfossils appear to be *in situ* and oriented in life position. When phosphatic laminae are examined in thin sections cut parallel to bedding (e.g. Fig. 6), the filaments (sheaths) are not preserved in life position, as would be expected in a well‐preserved microbial mat or stromatolite. Rather, they are scattered throughout the lamina with an essentially random orientation (as seen in the rose diagram in Fig. 6). Our supposition is that these microfossils are allochthonous and, as such, do not provide direct support of an *in situ* tufted mat composed of filamentous cyanobacteria as the reticulate miss builder. Thus, the question remains, how are the reticulate patterns formed and then preserved in positive relief?

It is interesting to note that the historical interpretation of these very structures began, without qualification, as raindrop impressions (Hinxman *in* Peach *et al*. 1907). Indeed, incontrovertible raindrop impressions do, in fact, occur throughout the shales of the Torridonian (Fig. 4E). This is not to say that the reticulate/tufted pattern of the Diabaig miss is not the result of microbe–sediment interaction, rather the particular raised pattern is not the direct result of the trapping and binding of sediment by filamentous cyanobacteria as a direct formative agent replicating the primary morphology of a tufted mat. Hinxman (*in* Peach *et al*. 1907) proposed that the Diabaig climate was so dry that muds impacted by raindrops were hardened prior to burial and compaction, thus preserving relief of the reticulate fabric. His supposition is reinforced by the occurrence of reticulate miss preserved within desiccation polygons at the Diabaig type locality (Fig. 5E). Here, multiple bedding surfaces are preserved with reticulate miss that is present both within the desiccation polygons (Fig. 5D) and covering over the in‐filled desiccation cracks (Fig. 5E, arrow). The microbial mats themselves must have periodically dried out, since their laminae are crosscut by mudcracks infilled with sediment and they subsequently overgrew those infilled cracks. This is seen at a millimetre scale in Fig. 5D which shows a close‐up of such an example of an infilled crack (c) which crosscuts the reticulate miss laminae. Thus, these microbially mediated sediments were directly exposed to the drying effects of air, just as Hinxman (*in* Peach *et al*. 1907) originally suggested.

Living microbial mats occurring adjacent to streams in the Highlands today also yield clues as to the possible genesis of reticulated structure in non‐marine settings. Figure 4B and D shows marginal algal mats along the Badachro River (0.5 km from its mouth) just after a period of sleet and rain. The impact of precipitation can be seen in the wetted subaerial mat, which shows both circular impacts and a reticulate pattern where impacts have occurred adjacent to each other (Fig. 4D). Intriguingly, as these mats dry out, this induced reticulate pattern is retained as illustrated in Fig. 4B. It is easy to envision that the combination of cohesive extracellular polymeric substances (eps) and precipitation impacts could cause the original sediment to retain this distinctive pattern.

The microbially derived cohesive eps which infused these sediments would have provided an additional means of retaining the sedimentary reticulum during subsequent burial and lithogenesis. In carbonate‐rich, stromatolite‐forming environments, eps is known to be sticky and cohesive (Reid *et al*. 2000), assisting in the trapping of oöids and other carbonate grains. A similar situation occurs in flat‐laminated microbial mats in siliciclastic environments (Franks and Stolz 2009). The cohesive function of microbially covered surfaces is probably independent of desiccation, the trapping and binding of sediment in modern microbial mats occurs both subaqueously and subaerially. Nevertheless, there is little doubt that the cohesive properties of eps as a biofilm component at Diabaig were important in helping to initially establish a sedimentary reticulum during burial and throughout diagenesis. These facts supplement Hinxman's observations and provide an enhanced version of his explanation for the retention of a reticulate surface pattern as evidence of raindrop impressions.

We propose that the reticulate miss at Diabaig was formed by a combination of biogenic and lithogenic events. Microbial mats growing on wetted surfaces produced cohesive eps, which formed a pervasive, cohesive biofilm in the upper millimetres of the sediment. Raindrops falling on drying surfaces produced reticulated patterns, which were retained by the biofilm–sediment layer. Subsequent burial and compaction, perhaps aided by early lithification, allowed for the retention of some of the primary relief in the sediment.

Direct evidence for this kind of microbially mediated model is observed in thin sections of phosphatic nodules that are found throughout the Diabaig Shale. These nodules are typically less than1 cm thick and up to several cm in diameter. Shale laminae which continue from surrounding rock into the nodules are more widely spaced inside the nodules, demonstrating that phosphatization occurred early in diagenesis, prior to full sedimentary compaction (Peach *et al*. 1907). Petrographic thin sections cut normal to the bedding of the reticulate miss do not support evidence of an *in situ* filamentous mat‐building community (Fig. 5A). Bedding‐parallel thin sections of the nodules might be expected to reveal the primary remains of the microbial mats along with their biologically degraded remains, but in most cases the microfossil remains appear fragmented and scattered as demonstrated in Figure 6. Thus, the organic faction that is retained in these rocks is probably more akin to a biofilm, than it is to a full blown microbial mat community.

### On the ecology of *Eohalothece* and its occurrence in reticulate miss

**Figure 7:**
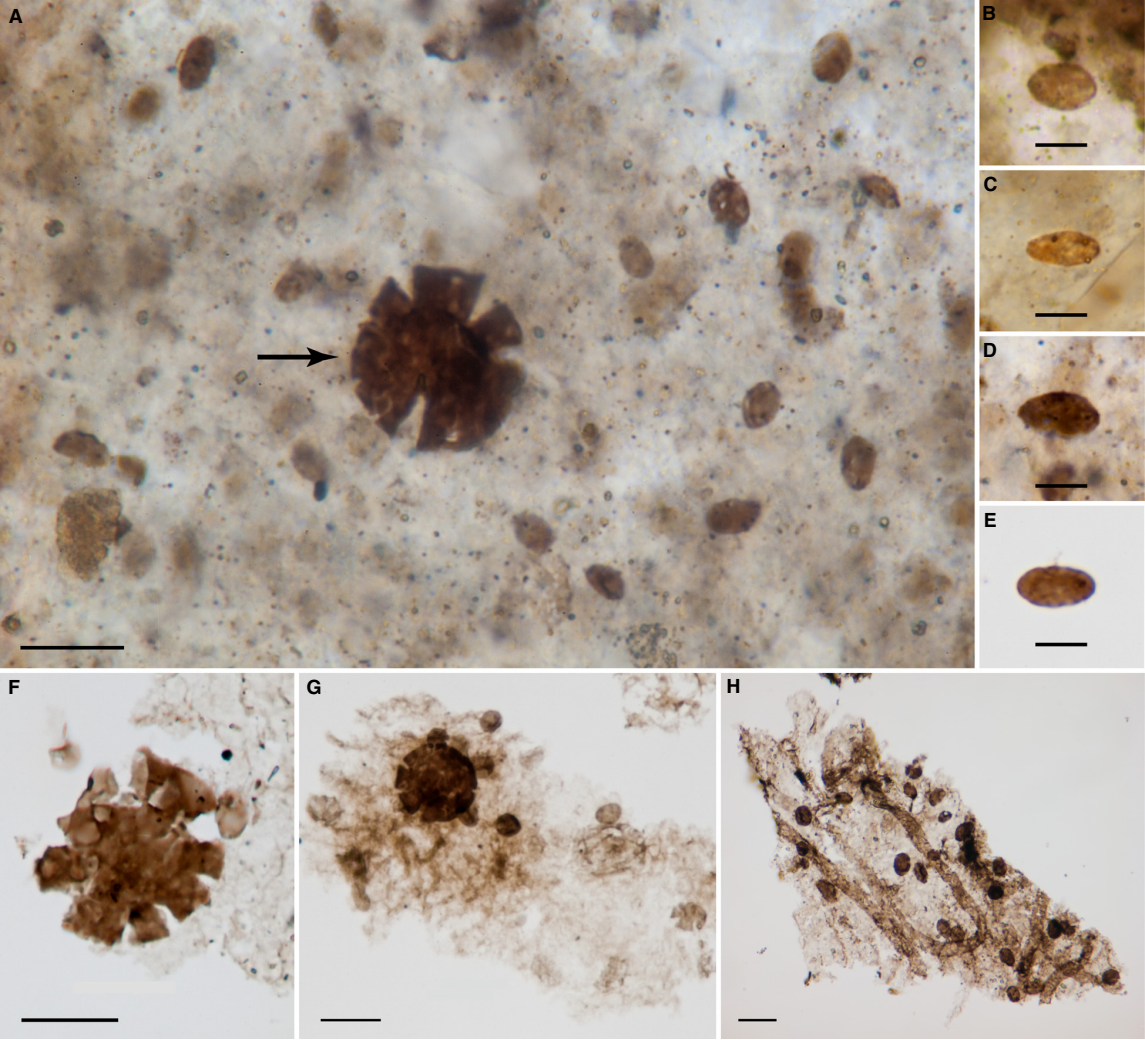
Evidence of *Eohalothece* found in association with biofilms. A, bedding‐parallel petrographic thin section in normal white light showing the distributed individual cells of *Eohalothece lacustrina* preserved *in situ* in a phosphate nodule, arrow points to a specimen of *Leiosphaeridia ternata* Timofeev, sample TOR08‐32, slide CW1C, type section of the Diabaig Formation, Lower Diabaig, Scotland. B, individual cell of *E. lacustrina* demonstrating elliptical shape from the thin‐section population shown in A. C, somewhat elongate individual cell of *E. lacustrina* from the thin‐section population shown in A. D, individual cell of *E. lacustrina* demonstrating its tapered elliptical form from the thin section population shown in A. E, individual cell of *E. lacustrina* demonstrating its tapered elliptical form, from maceration, Nonesuch Shale, sample NON09‐3B (paratype). F, macerated palynomorphs (*L. ternata* and *E. lacustrina*) associated with amorphous organic matter, which is a possible biofilm fragment, sample NON12‐22, Nonesuch Shale, well WPB5, 89 m depth. G, macerated palynomorphs (*L. ternata* and *E. lacustrina* paratype) associated with amorphous organic matter, possibly the remains of biofilm, sample NON09‐3B, Nonesuch Shale, well PC1, 93 m depth. H, microbial mat fragment with *E. lacustrina* and straight sheaths (similar to *Siphonophycus* sp.) embedded in amorphous organic matter, sample TOR09‐90B, Kinloch Fm, Ob Gauscavaig, Sleat Peninsula, Isle of Skye, Scotland. Scale bars represent 10 μm (A, F–H); and 5 μm (B–E). Colour online.

In Fig. 7A, a large population of *Eohalothece lacustrina* is distributed throughout a single organic‐rich lamina. The cells preserved in the lamina are more‐or‐less scattered randomly; they do not form tight associations with each other. Nor do they display evidence of cell division. A sample population of 52 cells from this lamina had a mean length of 6.0 μm with very low skewness (0.14). Unlike all the planktonic colonies isolated from macerated samples, this *in situ* population did not show a strong correlation with a logistic size distribution – the W/L vs Length plot had an r^2^ value of only 0.26, as compared to 0.65 to 0.9 for six measured populations recovered from maceration (p < 0.001; Table 1). So there is no evidence that this population represents a growing colony trapped *in situ*.

There are several sphaeromorph acritarchs distributed within this same layer of the thin section, most notable of which is *Leiosphaeridia ternata* Timofeev (Fig. 7A, arrow). The distribution of these cyst‐like forms is widely scattered and they appear to be allochthonous elements, perhaps planktonic fallout that had dropped into an organic‐rich benthos. Thus, our interpretation of this layer is that it represents a buried biofilm which preserved scattered elements of allochthonous origin, along with an i*n situ* population of the seemingly randomly oriented cells of *Eohalothece*. The question remains, however, as to the exact ecological nature of that *in situ* population – does it represent phytoplankton fallout, or a benthic, distributed growth form of *E. lacustrina*?

**Figure 8:**
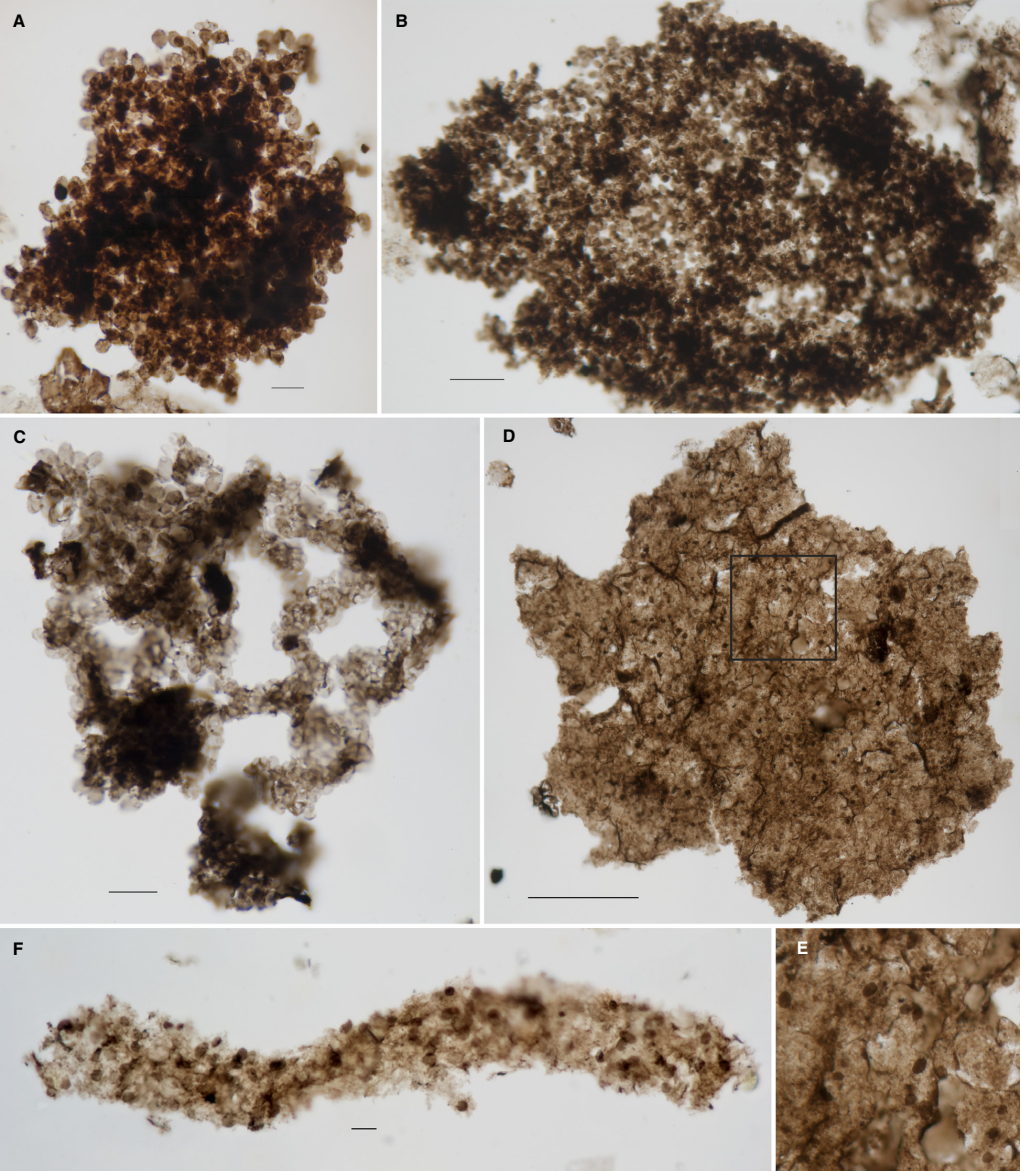
Evidence of *Eohalothece* as phytoplankton. A, large colony of *Eohalothece lacustrina* (paratype) with irregular globular overall form, sample NON09‐3B, Nonesuch Shale, well PC1, 93 m depth. B, large colony of *E. lacustrina* (paratype) with ovoid overall form, sample NON09‐3B, Nonesuch Shale, well PC1, 93 m depth. C, large colony of *E. lacustrina* displaying clathrate form similar to that found in planktonic freshwater species of *Microcystis* today, sample NON09‐24B, Nonesuch Shale, well WPB4, 54.5 m depth. D, distributed colony of *E. lacustrina* adhering to flat organic fragment; sample NON09‐25B, Nonesuch Shale, well WPB4, 59.5 m depth. E, detail of box in D, showing the specimens of *Eohalothece* adhering to the organic fragment, sample NON09‐25B, Nonesuch Shale, well WPB4, 59.5 m depth. F, oddly shaped fragment comprised of amorphous organic matter with an embedded population of *E. lacustrina*, sample NON09‐34C, Nonesuch Shale, well WPB4, 164 m depth. Scale bars represent 10 μm (A, C, F); 25 μm (B); and 100 μm (D). Colour online.

Populations of dispersed individuals of *Eohalothece* commonly occur in association with sheets of amorphous organic matter (Figs 7B–D, 8D–F). These are found in palynological preparations from localities throughout the Torridonian sequence and in cores of the Nonesuch Formation. In Figure 8D–E, dispersed cells of *E. lacustrina* are scattered over a cohesive organic layer. This layer does not appear to have retained any underlying cellular structure, so we interpret it to be the remnants of a once‐cohesive biofilm. In any case, the relation to *E. lacustrina* is similar to that seen in the *in situ* example illustrated in Figure 7A in which the cells appear to be randomly scattered over the surface. Sometimes cells of *E. lacustrina* are found in intimate association with amorphous (acellular) organic matter which appears to show some form of macroscopic structure, as is seen in Figure 7F, which shows a distinctly linear form. Far more common is the association of scattered individual cells of *E. lacustrina* with unstructured amorphous to diaphanous organic matter (Fig. 7B–D). It is somewhat remarkable that palynological preparations from the Nonesuch Formation include bits of organic groundmass that include both *Leiosphaeridia ternata* and *E. lacustrina* together (Fig. 7B–C) – just as seen *in situ* in phosphate (Fig. 7A). Occasionally, cells of *E. lacustrina* are found attached to or mixed in with sheaths of *Siphonophycus* (Fig. 7D). *Siphonophycus* is a well‐characterized Neoproterozoic cyanobacterium which forms microbial mats (Ruiji and Yin 2011). The direct co‐occurrence of *E. lacustrina* with *Siphonophycus* sheaths embedded in groundmass presents direct evidence of either ripped‐up mats or *in situ* mat/eps which has been disrupted during acid maceration processing.

### On the ecology of *Eohalothece* and its occurrence in colonies

While the occurrence of dispersed populations of individual cells of *Eohalothece* in benthic biofilms seems common, evidence of colonial forms of *Eohalothece* as plankton is also found in palynological preparations from the Nonesuch Formation. Populations of cells occur in large subspherical masses that range in size up to more than 300 μm (Fig. 8A–B). These larger colonial masses are similar in size and appearance to modern day *Microcystis*, species of which are common elements of the freshwater phytoplankton in a wide range of settings, from ephemeral ponds to large lakes (Desikachary 1959). One of the more distinctive features of extant *Microcystis* and related planktonic chroococcalean cyanobacteria is the clathrate colonial form, in which multitudinous strands of cells form a three‐dimensional, netlike gross shape. Figure 8C shows an example of the clathrate colonial form in *E. lacustrina* recovered from a macerated sample of the Nonesuch Formation. In modern settings, this gross population structure is found associated only with planktonic colonies. As such, its recovery in the Nonesuch shales provides convincing support for the case that *E. lacustrina* is both a chroococcalean cyanobacterium and that it was capable, in colonial form, of floating in the water column of the earliest Neoproterozoic lakes.

### *Eohalothece* and phosphogenesis: a new hypothesis

The shales of the Torridonian have long been characterized by the presence of phosphatic nodules (Peach *et al*. 1907). In some of the thin sections, we have examined whether there is an abundance of phosphate in association with *Eohalothece lacustrina* (Fig. 7A). In fact, the same bedding plane in which these specimens are preserved gradually grades into a zone of granular phosphate (online Supporting Information, Fig. S2). Wacey *et al*. (2014) were able to demonstrate an intimate association of the phosphatic mineral francolite with microfossils within Torridonian phosphates. It is most often clay minerals that are in immediate contact with remaining structural organic matter, such as cell walls, and phosphate is most often preserved externally to this carbon–clay complex. They showed, for example, that presumed sheaths of fossil cyanobacteria (Wacey *et al*. 2014, fig. 4) were encased in phosphate with clays occupying cell interiors. The topological relationship of phosphate to eukaryotic microfossils was similar, with francolite encasing clay‐rich interiors and organic walls. But this characterization of clays on the inside and phosphate on the outside was not absolute, and Wacey *et al*. (2014, fig. 8) also demonstrated the presence of individual francolite crystals formed inside a prokaryotic microfossil fossil cell. Their hypothesis as to the ultimate source of P_i_ was nutrient enrichment from runoff which would have caused blooms in both planktonic and benthic settings. Francolite precipitation was promoted by the release of P_i_ previously adsorbed onto iron oxides and hydroxides in combination with release of P_i_ from decaying organic matter. Both of these mechanisms were promoted by reducing conditions during burial and authigenic mineralization. The new model, presented below, adds to that of Wacey *et al*. (2014) by providing an additional biochemical argument for the delayed release of P_i_ and its biological loss (via authigenic mineralization of francolite) in the Torridonian lacustrine ecosystem.

*Microcystis aeruginosa*, along with *Planktothrix*,* Anabaena* and other cyanobacteria, are known to be prolific producers of microcystins (MCs), non‐ribosomal polypeptides that function as phosphatase inhibitors (Rantala *et al*. 2004). Inside the cell, phosphatases mediate dephosphorylation reactions. These are hydrolytic reactions that release inorganic phosphate (PO_4_^3−^) from bound organic phosphate. Therefore, MCs, as produced by cyanobacteria, effectively function to prevent the release of inorganic phosphate from its organically bound form. The toxic effects of cyanobacterial blooms in lakes have been known for well over a century (Francis 1878), and the association of *Microcystis aeruginosa* with toxicity in lakes is also well established (Ashworth and Mason 1946; Elleman *et al*. 1978). Microcystin‐based toxicity has been evoked as a causal agent in both mass extinction (Castle and Rodgers 2009) and mass mortality studies (Braun and Pfeiffer 2002). Von Koenigswald *et al*. (2004) were able to determine that seasonal blooms of cyanobacteria were likely to be responsible for mass deaths of turtles and horses as well as birds and bats in the Eocene Messel Lake. It has been established that the microcystin synthetase gene set is primitive (Rantala *et al*. 2004), so the palaeobiological applicability of the toxic effects of cyanobacteria is perhaps warranted in those cases where cyanobacteria were known to be present, as is the case with the Messel ecosystem in Germany (von Koenigswald *et al*. 2004). It seems highly likely that the cyanobacteria living in the Torridonian palaeoecosystem were capable of producing microcystins.

If active extracellular MCs were to block dephosphorylation of organic phosphates in eps, for example, they would act to effectively trap phosphorus in environmental eps by preventing the release of inorganic phosphate (P_i_) back into the environment. This inference is speculative, as we do not know whether MCs did function this way in settings outside the cell. But given that MCs actively inhibit intercellular phosphorylation (Codd 1995) and that these compounds retain extracellular functionality (Wert *et al*. 2014), it seems reasonable to suggest that they may have acted to suppress hydrolysis of organic phosphates.

A second consideration is that the phosphatase inhibitory function of MCs acted to suppress P_i_ release during bacterial decay of organic phosphate. This functionality, the suppression of protein phosphatases in bacteria, has been documented in modern freshwater settings (MacKintosh *et al*. 1990; Codd 1995). Thus, the presence of MCs today acts to suppress remobilization of P_i_ in organic‐rich bottom sediments. In fact, any delay in the release of phosphorous from its organically bound form would promote the burial and eventual trapping of phosphorous in sediment. The mechanism for the precipitation of mineralized phosphate in lacustrine settings involves a two‐step process: (1) the stabilization, retention and subsequent burial of organic phosphorous; and (2) the delayed release of P_i_ from its organically bound form to act as a source of dissolved inorganic P. The possibility of MCs assisting in these processes, based on their properties as phosphatase inhibitors, seems quite probably given their ability to survive and remain chemically active as exoenzymes in the extracellular environment.

As pollutants, MCs are toxic for a wide variety of organisms, including humans. This has stimulated research on the relations between MC production by cyanobacteria and environmental conditions associated with cyanobacterial blooms in nature. We can use these studies to gain an understanding of which environmental conditions are conducive to the excess production of MCs and to infer something about possible environmental conditions during Torridonian time. For example, the production of MCs within the cells of cyanobacteria has been shown to be stimulated by the presence of intracellular Fe^2+^ (Utkilen and Gjølme 1995). It has been demonstrated experimentally that intracellular MCs are increasingly released during oxidation – MCs can survive outside the cell and they can remain active under oxidizing conditions (Wert *et al*. 2014). *Microcystis aeruginosa*, when marked as a producer of toxic levels of MCs, appears to flourish under conditions of nitrogen limitation. MCs are more abundant in lakes with less total nitrogen (TN) (Graham *et al*. 2004; Rinta‐Kanto *et al*. 2009), and Watanabe and Oishi (1985) found that MCs increased in cells grown under conditions of 1/10 and 1/20 normal growth levels of nitrogen. More recently, Orihel *et al*. (2012) demonstrated specifically that high levels of MCs occur in lakes with low N:P, and maximum concentrations occur at mass N:P ratios below 23. As *M. aeruginosa* is a non‐nitrogen fixing cyanobacterium, it seems that the production of MCs is not specifically linked to settings which require cyanobacteria to fix their own nitrogen, even though it appears that MCs are associated with conditions of poor nitrogen availability.

The significance of this observation has to do with the relative balance of nutrient availability in the Torridonian palaeoecosystem and its relation to the presence of early diagenetic phosphate preserved in that system. Permineralized phosphate in sedimentary rock under these circumstances indicates a loss of biologically available phosphate within a palaeoecosystem. The persistence of phosphatic laminae and nodules throughout the Diabaig shales is evidence of a dynamic loss of P_i_ from the overlying water column during the sedimentation of the Diabaig muds and silts. In spite of this exodus of P_i_ from the Diabaig microbial community, the production of organic matter (as indicated by the richness of the microflora and distributed refractory organic matter throughout the formation) indicates persistent biological productivity throughout. This indicates that the loss of P_i_ was not a deterrent to normal levels of growth and productivity, which, in turn, implies a condition of nitrogen or carbon limitation. In this regard, it is curious that in living species of cyanobacteria, as indicated above, the release of MCs is enhanced under conditions of nitrogen limitation. Overall, this suggests that the Torridonian lacustrine ecosystem may have been nitrogen limited.

Previous genetic models of Precambrian and Cambrian phosphate deposition have assumed that P_i_ accumulates in sediment as a consequence of excess biological productivity brought about by the delivery of high levels of P_i_ as a biological nutrient. P_i_ as a source of mineralized phosphate is more‐or‐less thought of as leakage from associated buried organic matter. This can occur in the form of runoff in coastal (She *et al*. 2013) or shallow marine setting or in upwelling zones associated with the delivery of deep ocean water to continental shelf margins (Xiao and Knoll 1999). Clearly, oceanic upwelling was not a possible source of phosphorous in the lacustrine setting of the Diabaig muds, so P_i_ in the Torridonian palaeoecosystem must have been delivered via runoff and recycled within the local biota.

Biologically mediated release of P_i_ from environmentally bound organic phosphate has been proposed for many systems, both ancient and extant (Föllmi 1996). For example, Barale *et al*. (2013), in a study of Mesozoic pelloidal ironstones, argued that Fe‐reducing bacteria induced precipitation of mineralized phosphate through the release of P_i_ from both organic phosphate and reduced Fe oxides which are capable of retaining P_i_. This was based, in part, on their observation of presumed phosphatized bacteria in intimate association with the surfaces of phosphatized oöids (although Föllmi (1996) earlier noted that the mineral form of francolite itself is rod‐shaped and could be mistaken for a bacterial form). They proposed the release of bacterial phosphatases as a mechanism causing as the subsequent release of P_i_ as a source for phosphate mineralization.

Hubert (2005), utilizing a detailed study of phosphate mineralization in relation to organic remains, demonstrated that for the Doushantuo phosphorites, biological retention of P_i_ took place at the sediment–water interface and that microbial mats were responsible for trapping P_i_ and preventing its release back into the water column. She *et al*. (2014) demonstrated a very close association of fossil cyanobacteria, especially *Myxococcoides*, with granular phosphorite deposition in the Doushantuo Formation. The conclusions of these studies of phosphate deposition in Neoproterozoic marine settings are not incompatible with the model presented here for non‐marine settings; however, the applicability of the MC‐enhanced retention of Pi remains an unknown at this point as the production of MCs in marine settings is not well studied.

In summary, our model provides a specific mechanism for the retention of P_i_ in the form of organic phosphate within organic‐rich sediments. Phosphorous trapped in this way was not recycled back into the biosphere, but instead accumulated in the sediment as buried organic phosphate. The presence of MCs associated with benthic microbial mats/biofilms acted to delay the release immediate of P_i_ back into the environment, either through the inhibition of bacterial phosphatases during respiration, or through the suppression of phosphatase activity during the breakdown of eps. The buried organic phosphate that occurred in this way would have released P_i_ only as diagenesis proceeded, eventually reaching concentrations sufficient to precipitate as francolite.

If phosphogenesis in Precambrian lakes (and some marine settings, for that matter) is so closely tied to cyanobacterial physiology, why are phosphatic deposits lacking in the Nonesuch lake system and especially given the abundance of *E. lacustrina* in the Nonesuch cores? The answer probably lies in specific differences in the environmental ecology of the two lake systems. We have already inferred that the Torridonian lakes may have been nitrogen limited in terms of nutrient input. Phosphorous input from weathering and runoff is tied to source terrain: the Torridonian lakes sit directly on Lewisian gneisses which provided a more that adequate source of P_i_; the Nonesuch lacustrine sediments were derived, in part, from volcanic terrain. Thus, the trophic ecology of the Nonesuch lake was more likely to have been phosphorous limited than nitrogen limited. This is speculative, of course, but, at the very least, comparison of authigenic mineral formation between the two deposits might provide direct insight into ecological heterogeneities between the two deposits.

## Conclusion

Micropalaeontological studies of lacustrine deposits from the Torridon Group and the Nonesuch Formation provide a snapshot of non‐marine life at the very end of the Mesoproterozoic. With the description of the new taxon *Eohalothece,* there is now direct evidence of cyanobacteria occupying both benthic and planktonic settings in freshwater habitats at this time, although it is not clear that *Eohalothece* necessarily grew in bottom sediment, there is evidence that they did actively grow to form large, planktonic colonies. While it is likely that numerous forms of eukaryotic algae co‐occur in these deposits in the form of various spherical to subspherical cysts, there are no specific characters displayed in these microfossils that link any specimens unambiguously to the green algae. Other lines of evidence indicate that the chlorophytes must have evolved by this time (Knoll *et al*. 2007), so one might assume that many of the small sphaeromorphs found throughout these sections are the remains of eukaryotic chlorophytes, but that the green algae had yet to fully diversify as freshwater plankton. However, the large globular colonies and clathrate forms associated with *Eohalothece* do match adaptive responses to the planktonic habitat seen in cyanobacteria today. This combination of a lack of morphological characters unique to the chlorophyta along with specific gross morphologies associated with cyanobacterial populations leads to the conclusion that the cyanobacteria were a significant to dominant component of the primary producers in terrestrial settings at 1 Ga (Wellman and Strother 2015).

The intimate physical relationship seen in phosphate deposition and *in situ* populations of *Eohalothece* in the Diabaig Formation favours a biologically driven cause to the formation of early diagenetic phosphatic nodules. The release of MCs by cyanobacteria such as *Microcystis aeruginosa* in lakes today provides the basis for speculating that a similar condition in the Torridonian lakes could have contributed to the retention of organic phosphate during burial, with the subsequent release and mineralization of P_i_ occurring during the early phases of diagenesis. This mechanism would have effectively removed phosphorus from biological recycling. This model has the side effect of predicting that the Torridonian lakes ecosystems were nitrogen limited. It is interesting to note that some of the granular phosphorites of the Doushantuo Formation are also intimately associated with blooms of cyanobacteria (She *et al*. 2013, 2014), although these formed in marine settings for which there is little neontological evidence of microcystin production.

Lastly, the palaeoecology of miss associated with the basal lacustrine units in the Diabaig Formation in the Torridon Group can be interpreted in terms of their associated microfossils. Within the laminated shales and siltstones of the Diabaig, filamentous microfossils generally appear allochthonous without signs of being preserved in life position. In general, the scattered and disoriented arrangement of filaments appears random in phosphatic sections, too. Occasionally, bedding‐parallel thin sections of phosphatic nodules do reveal what appear to be eps‐rich biofilms that preserved populations of scattered individuals. Such samples are not characterized by filamentous mat‐builders, as is the model for stromatolite builders in coeval carbonate settings, but by somewhat evenly distributed cells of the new genus, *Eohalothece*. When combined with a long history of observations of primary sedimentary features associated with raindrop impressions in the Torridonian sequence (Hinxman *in* Peach *et al*. 1907), it seems apparent that the reticulated miss at Diabaig is not due to the trapping and binding of sediment by filamentous cyanobacteria, but rather reflects raindrop impacts on subaerially exposed microbial slime, whose surficial patterning was retained by early cementation of organically bound microfabrics.

## Supporting information


**Fig. S1.** Three populations of *E. lacustrina* with their corresponding size data.

**Fig. S2.** Images from thin section of a phosphatic nodule cut parallel to bedding.

## References

[pala12212-bib-0001] Ashworth, C. T. and Mason, M. F. 1946 Observations of the pathological changes produced by a toxic substance present in blue‐green algae *(Microcystis aeruginosa)* . American Journal of Pathology, 22, 369–383. 21018103

[pala12212-bib-0101] Barale, L. , D'Atri, A. and Martire, L. 2013 The role of microbial activity in the generation of Lower Cretaceous mixed FE‐oxide‐phosphate ooids from the Provencal Domain, French Maritime Alps. Journal of Sedimentary Research, 83, 196–206.

[pala12212-bib-0002] Barghoorn, E. S. , Meinschein, W. G. and Schopf, J. W. 1965 Paleobiology of a Precambrian shale: geology, organic geochemistry, and paleontology are applied to the problem of detection of ancient life. Science, 148, 461–472. 17842832 10.1126/science.148.3669.461

[pala12212-bib-0005] Braun, A. and Pfeiffer, T. 2002 Cyanobacterial blooms as the cause of a Pleistocene large mammal assemblage. Paleobiology, 28, 139–154.

[pala12212-bib-0007] Browne, K. M. , Golubic, S. and Seong‐Joo, L. 2000 Shallow marine microbial carbonate deposits 233–249. *In* Riding R. E. and Awramik S. M. (eds). Microbial sediments. Springer‐Verlag, Berlin, Heidelberg, 331 pp.

[pala12212-bib-0009] Callow, R. H. T. , Battison, L. and Brasier, M. D. 2011 Diverse microbially induced sedimentary structures from 1Ga lakes of the Diabaig Formation, Torridon Group, northwest Scotland. Sedimentary Geology, 239, 117–128.

[pala12212-bib-0010] Castle, J. W. and Rodgers, J. H. 2009 Hypothesis for the role of toxin‐producing algae in Phanerozoic mass extinctions based on evidence from the geologic record and modern environments. Environmental Geosciences, 16, 1–23.

[pala12212-bib-0011] Cloud, P. and Germs, A. 1971 New pre‐Paleozoic nannofossils from the Stoer Formation (Torridonian), northwest Scotland. Geological Society of America Bulletin, 82, 3469–3474.

[pala12212-bib-0012] Codd, G. A. 1995 Cyanobacterial toxins: occurrence, properties and biological significance. Water Science and Technology, 32, 149–156.

[pala12212-bib-0013] Cumming, V. M. , Poulton, S. W. , Rooney, A. D. and Selby, D. 2013 Anoxia in the terrestrial environment during the late Mesoproterozoic. Geology, 41, 583–586.

[pala12212-bib-0016] Davis, D. W. and Paces, J. B. 1990 Time resolution of geologic events on the Keweenaw Peninsula and implications for development of the Midcontinent Rift System. Earth and Planetary Science Letters, 97, 54–64.

[pala12212-bib-0017] Desikachary, D. V. 1959 Cyanophyta. Indian Council of Agricultural Research, Bombay, 686 pp.

[pala12212-bib-0018] Downie, C. 1961 The so‐called spores of the Torridonian. Proceedings of the Geological Society of London, 1600, 127–128.

[pala12212-bib-0019] Downie, C. 1973 Observations on the nature of the acritarchs. Palaeontology, 16, 239–259.

[pala12212-bib-0021] Elleman, T. C. , Falconer, I. R. , Jackson, A. R. B. and Runnegar, M. T. 1978 Isolation, characterization and pathology of the toxin from a *Microcystis aeruginosa (=Anacystis cyanea)* bloom. Australian Journal of Biological Sciences, 31, 209–218. 103520 10.1071/bi9780209

[pala12212-bib-0022] Elmore, R. D. , Milavec, G. J. , Imbus, S. W. and Engel, M. H. 1989 The Precambrian Nonesuch Formation of the North American Mid‐Continent Rift, sedimentology and organic geochemical aspects of lacustrine deposition. Precambrian Research, 43, 191–213.

[pala12212-bib-0024] Föllmi, K. B. 1996 The phosphorus cycle, phosphogenesis and marine phosphate‐rich deposits. Earth‐Science Reviews, 40, 55–124.

[pala12212-bib-0025] Francis, G. 1878 Poisonous Australian lake. Nature (London), 18, 11–12.

[pala12212-bib-0026] Franks, F. and Stolz, J. 2009 Flat laminated microbial mat communities. Earth‐Science Reviews, 96, 163–172.

[pala12212-bib-0027] Garcia‐Pichel, F. , Nübel, U. and Muyzer, G. 1998 The phylogeny of unicellular, extremely halotolerant cyanobacteria. Archives of Microbiology, 169, 469–482. 9575232 10.1007/s002030050599

[pala12212-bib-0028] Graham, J. L. , Jones, J. R. , Jones, S. B. , Downing, J. A. and Clevenger, T. E. 2004 Environmental factors influencing microcystin distribution and concentration in the midwestern United States. Water Research, 38, 4395–4404. 15556214 10.1016/j.watres.2004.08.004

[pala12212-bib-0069] van Gremberghe, I. , Leliaert, F. , Mergeay, J. , Vanormelingen, P. , van der Gucht, K. , Debeer, A.‐E. , Lacerot, G. , de Meester, L. and Vyverman, W. 2011 Lack of phylogeographic structure in the freshwater cyanobacterium *Microcystis aeruginosa* suggests global dispersal. PloS One, 6, e19561. 21573169 10.1371/journal.pone.0019561 PMC3088681

[pala12212-bib-0029] Hieshima, G. and Pratt, L. 1991 Sulfur/carbon ratios and extractable organic matter of the Middle Proterozoic Nonesuch Formation, North American Midcontinent Rift. Precambrian Research, 54, 65–79.

[pala12212-bib-0030] Ho, E. S. , Meyers, P. A. and Mauk, J. L. 1990 Organic geochemical study of mineralization in the Keweenawan Nonesuch Formation at White Pine, Michigan. Organic Geochemistry, 16, 229–234.

[pala12212-bib-0106] Hofmann, H. J. 1976 Precambrian microflora, Belcher Islands, Canada: significance and systematics. Journal of Paleontology, 50, 1040–1073.

[pala12212-bib-0031] Hubert, B. 2005 Microbially mediated phosphatization in the Neoproterozoic Doushantuo lagerstätte, South China. Bulletin de la Société de France, 176, 355–361.

[pala12212-bib-0033] Knoll, A. H. , Summons, R. E. , Waldbauer, J. R. and Zumberge, J. E. 2007 The geological succession of primary producers in the oceans 133–163. *In* Falkowski P. G. and Knoll A. H. (eds). Evolution of primary producers in the sea. Academic Press, Burlington, MA, 441 pp.

[pala12212-bib-0034] Koenigswald, W. Von , Braun, A. and Pfeiffer, T. 2004 Cyanobacteria and seasonal death: a new taphonomic model for the Eocene Messel lake. Paläontologische Zeitschrift, 78, 417–424.

[pala12212-bib-0102] Kützing, F. T. 1845–1849. Tabulae phycologicae oder Abbildungen der Tange, *vol. 1* Nordhausen, 1–54.

[pala12212-bib-0103] Lo, S. C. 1980 Microbial fossils from the lower Yudoma suite, earliest Phanerozoic, eastern Siberia. Precambrian Research, 13, 109–166.

[pala12212-bib-0035] Mackintosh, C. , Beattie, K. A. , Klumpp, S. , Cohen, P. and Codd, G. A. 1990 Cyanobacterial microcystin‐LR is a potent and specific inhibitor of protein phosphatases 1 and 2A from both mammals and higher plants. FEBS Letters, 264, 187–192. 2162782 10.1016/0014-5793(90)80245-e

[pala12212-bib-0036] Meinschein, W. G. , Barghoorn, E. S. and Schopf, J. W. 1964 Biological remnants in a Precambrian sediment. Science, 145, 262–263. 17833033 10.1126/science.145.3629.262

[pala12212-bib-0037] Moore, L. R. , Moore, J. R. M. and Spinner, E. 1969 A geomicrobiological study of the Pre‐Cambrian Nonesuch Shale. Proceedings of the Yorkshire Geological Society, 37, 351–394.

[pala12212-bib-0038] Nägeli, C. 1848 Gattungen Einzelliger Algen, Physiologisch und Systematisch Bearbeitet. F. Schulthess, Zürich, 139 p.

[pala12212-bib-0039] Naumova, S. N. and Pavlovsky, G. V. 1961 The discovery of plant remains (spores) in the Torridonian shales of Scotland. Doklady Akademii Nauk SSSR, 141, 181–182.

[pala12212-bib-0104] Nyberg, A. V. and Schopf, J. W. 1984 Microfossils in stromatolitic cherts from the upper Proterozoic Min'yar Formation, Southern Ural Mountains, USSR. Journal of Paleontology, 58, 738–772. 11541991

[pala12212-bib-0040] Orihel, D. M. , Bird, D. F. , Brylinsky, M. , Huirong, C. , Derek, D. B. , Huang, D. Y. , Alessandra, G. , Kinniburgh, D. , Kling, H. , Kotak, B. G. , Leavitt, P. R. , Nielsen, C. C. , Reedy, S. K. , Rooney, R. C. , Watson, S. B. , Zurawell, R. W. and Vinebrooke, R. D. 2012 High microcystin concentrations occur only at low nitrogen‐to‐phosphorus ratios in nutrient‐rich Canadian lakes. Canadian Journal of Fisheries and Aquatic Sciences, 69, 1457–1462.

[pala12212-bib-0105] Parnell, J. , Mark, D. , Fallick, A. E. , Boyce, A. and Thakrey, S. 2011 The age of the Mesoproterozoic Stoer Group sedimentary and impact deposits, NW Scotland. Journal of the Geological Society, 168, 349–358.

[pala12212-bib-0041] Peach, B. N. , Horne, J. , Gunn, W. , Hinxman, L. W. and Teall, J. J. H. 1907 The geological structure of the north‐west Highlands of Scotland. Geikie A. (ed.) Memoirs of the Geological Survey of Great Britain, His Majesty's Stationery Office, Glasgow, 668 pp.

[pala12212-bib-0042] Peat, C. J. 1984 Comments on some of Britain's oldest microfossils. Journal of Micropalaeontology, 3, 65–71.

[pala12212-bib-0043] Peat, C. J. and Diver, W. L. 1982 First signs of life on Earth. New Scientist, 95, 776–781.

[pala12212-bib-0107] Pedentchouk, N. , Freeman, K. H. , Harris, N. B. , Clifford, D. J. and Grice, K. 2004 Sources of alkylbenzenes in Lower Cretaceous lacustrine source rocks, West African rift basins. Organic Geochemistry, 35, 33–45.

[pala12212-bib-0045] Pedentchouk, N. , Summons, R. E. and Hieshima, G. B. 1991 Sterane and triterpane biomarkers in the Precambrian Nonesuch Formation, North American Midcontinent Rift. Geochimica et Cosmochimica Acta, 55, 911–916.

[pala12212-bib-0046] Prave, A. R. 2002 Life on land in the Proterozoic: evidence from the Torridonian rocks of northwest Scotland. Geology, 30, 811–814.

[pala12212-bib-0047] Rantala, A. , Fewer, D. P. , Hisbergues, M. , Rouhiainen, L. , Vaitomaa, J. , Börner, T. and Sivonen, K. 2004 Phylogenetic evidence for the early evolution of microcystin synthesis. Proceedings of the National Academy of Sciences of the USA, 101, 568–573. 14701903 10.1073/pnas.0304489101 PMC327188

[pala12212-bib-0048] Reid, R. P. , Visscher, P. T. , Decho, A. W. , Stolz, J. F. , Bebout, B. M. , Dupraz, C. , Macintyre, I. G. , Paerl, H. W. , Pinckney, J. L. , Prufert‐Bebout, L. , Steppe, T. F. and Desmarais, D. J. 2000 The role of microbes in accretion, lamination and early lithification of modern marine stromatolites. Nature, 406, 989–992. 10984051 10.1038/35023158

[pala12212-bib-0049] Retallack, G. J. and Mindszenty, A. 1994 Well preserved late Precambrian Paleosols from Northwest Scotland. Journal of Sedimentary Research, 64, 264–281.

[pala12212-bib-0050] Rinta‐Kanto, J. M. , Konopko, E. A. , Debruyn, J. M. , Bourbonniere, R. A. , Boyer, G. L. and Wilhelm, S. W. 2009 Lake Erie *Microcystis*: relationship between microcystin production, dynamics of genotypes and environmental parameters in a large lake. Harmful Algae, 8, 665–673.

[pala12212-bib-0051] Ruiji, C. and Yin, L. 2011 Microbiota and microbial mats within ancient stromatolites in South China 65–86. *In* Tewari V. and Seckbach J. (eds.) Stromatolites: interaction of microbes with sediments. Cellular Origin, Life in Extreme Habitats and Astrobiology, 18, Springer, 752 pp.

[pala12212-bib-0052] Ruiz, J. , Jones, L. M. and Kelly, W. C. 1984 Rubidium‐strontium dating of ore deposits hosted by Rb‐rich rocks, using calcite and other common Sr‐bearing minerals. Geology, 12, 259–262.

[pala12212-bib-0053] Samylina, O. S. and Gerasimenko, L. M. 2011 Fossilization of the cells of natronophilic endoevaporite cyanobacterium ‘*Euhalothece natronophila*’ in a modelling system. Microbiology, 80, 525–534. 22073554

[pala12212-bib-0108] Schopf, J. W. 1968 Microflora of the Bitter Springs Formation, late Precambrian, central Australia. Journal of Paleontology, 42, 651–688.

[pala12212-bib-0055] Selley, R. C. 1965 The Torridonian succession on the islands of Fladday, Raasay, and Scalpay, Inverness‐shire. Geological Magazine, 102, 361–369.

[pala12212-bib-0056] She, Z.‐B. , Strother, P. , McMahon, G. , Nittler, L. R. , Wang, J. , Zhang, J. , Sang, L. , Changqian, M. A. and Papineau, D. 2013 Terminal Proterozoic cyanobacterial blooms and phosphogenesis documented by the Doushantuo granular phosphorites I: in situ micro‐analysis of textures and composition. Precambrian Research, 235, 20–35.

[pala12212-bib-0057] She, Z.‐B. , Strother, P. and Papineau, D. 2014 Terminal Proterozoic cyanobacterial blooms and phosphogenesis documented by the Doushantuo granular phosphorites II: microbial diversity and C isotopes. Precambrian Research, 251, 62–79.

[pala12212-bib-0058] Stanier, R. Y. , Sistrom, W. R. , Hansen, T. A. , Whitton, B. A. , Castenholz, R. W. , Pfennig, N. , Gorlenko, V. N. , Kondratieva, E. N. , Eimhjellen, K. E. , Whittenbury, R. , Gherna, R. L. and Trüper, H. G. 1978 Proposal to place the nomenclature of the Cyanobacteria (blue‐green algae) under the rules of the international code of nomenclature of bacteria. International Journal of Systematic and Evolutionary Microbiology, 28, 335–336.

[pala12212-bib-0059] Stewart, A. D. 2002 The later Proterozoic Torridonian rocks of Scotland: their sedimentology, geochemistry and origin. Memoirs of the Geological Society of London, 24, 1–136.

[pala12212-bib-0060] Stewart, A. D. and Parker, A. 1979 Palaeosalinity and environmental interpretation of red beds from the late Precambrian (‘Torridonian’) of Scotland. Sedimentary Geology, 22, 229–241.

[pala12212-bib-0061] Strother, P. K. 1994 Sedimentation of palynomorphs in rocks of pre‐Devonian age 489–502. *In* Traverse, A. (ed.) Sedimentation of organic particles. Cambridge University Press, 543 pp.

[pala12212-bib-0062] Strother, P. K. , Knoll, A. H. and Barghoorn, E. S. 1983 Micro‐organisms from the late Precambrian Narssârssuk Formation, north‐western Greenland. Palaeontology, 26, 1–32.

[pala12212-bib-0063] Strother, P. K. , Battison, L. , Brasier, M. D. and Wellman, C. H. 2011 Earth's earliest non‐marine eukaryotes. Nature, 473, 505–509. 21490597 10.1038/nature09943

[pala12212-bib-0064] Suszek, T. 1997 Petrography and sedimentation of the middle Proterozoic (Keweenawan) Nonesuch Formation, western Lake Superior region, Midcontinent Rift System. Geological Society of America Special Papers, 312, 195–210.

[pala12212-bib-0065] Thuret, G. 1875 Essai de classification des Nostochinées. Annales Sciences Naturelle (Botanique), 6, 372–382.

[pala12212-bib-0066] Turnbull, M. J. M. , Whitehouse, M. J. and Moorbath, S. 1996 New isotopic age determinations for the Torridonian, NW Scotland. Journal of the Geological Society, 153, 955–964.

[pala12212-bib-0067] Upfold, R. L. 2009 Tufted microbial (cyanobacterial) mats from the Proterozoic Stoer Group, Scotland. Geological Magazine, 121, 351–355.

[pala12212-bib-0068] Utkilen, H. and Gjølme, N. 1995 Iron‐stimulated toxin production in *Microcystis aeruginosa* . Applied and Environmental Microbiology, 61, 797–800. 7574617 10.1128/aem.61.2.797-800.1995 PMC167340

[pala12212-bib-0070] Wacey, D. , Saunders, M. , Roberts, M. , Menon, S. , Green, L. , Kong, C. , Culwick, T. , Strother, P. and Brasier, M. D. 2014 Enhanced cellular preservation by clay minerals in 1 billion‐year‐old lakes. Scientific Reports, 4, 5841. doi:10.1038/srep05841 25068404 10.1038/srep05841 PMC5376168

[pala12212-bib-0071] Watanabe, M. F. and Oishi, S. 1985 Effects of environmental factors on toxicity of a cyanobacterium (*Microcystis aeruginosa)* under Culture Conditions. Applied and Environmental Microbiology, 49, 1342–1344. 3923932 10.1128/aem.49.5.1342-1344.1985 PMC238555

[pala12212-bib-0072] Wellman, C. H. and Strother, P. K. 2015 The terrestrial biota prior to the origin of land plants (embryophytes): a review of the evidence. Palaeontology, 58, 601–627.

[pala12212-bib-0073] Wert, E. C. , Korak, J. A. , Trenholm, R. A. and Rosario‐Ortiz, F. L. 2014 Effect of oxidant exposure on the release of intracellular microcystin, MIB, and geosmin from three cyanobacteria species. Water Research, 52, 251–259. 24289950 10.1016/j.watres.2013.11.001

[pala12212-bib-0074] Wettstein, R. 1924 Handbuch der Systematischen Botanik, *Vol. 1* Franz Deuticke, Leipzig, 467 pp.

[pala12212-bib-0075] Woese, C. R. and Fox, G. E. 1977 Phylogenetic structure of the prokaryotic domain: the primary kingdoms. Proceedings of the National Academy of Sciences of the USA, 74, 5088–5090. 270744 10.1073/pnas.74.11.5088 PMC432104

[pala12212-bib-0076] Woese, C. R. , Kandler, O. and Wheelis, M. L. 1990 Towards a natural system of organisms: proposal for the domains Archaea, Bacteria, and Eucarya. Proceedings of the National Academy of Sciences of the USA, 87, 4576–4579. 2112744 10.1073/pnas.87.12.4576 PMC54159

[pala12212-bib-0077] Xiao, S. and Knoll, A. H. 1999 Fossil preservation in the Neoproterozoic Doushantuo phosphorite lagerstätte, South China. Lethaia, 32, 219–240. 11543524 10.1111/j.1502-3931.1999.tb00541.x

[pala12212-bib-0109] Zhang, Z. 1982 Upper Proterozoic microfossils from Summer Isles, N. W. Scotland. Palaeontology, 25, 443–460.

[pala12212-bib-0110] Zhang, Y. 1988 Proterozoic stromatolitic micro‐organisms from Hebei, North China: cell preservation and cell division. Precambrian Research, 38, 165–175.

