# Supplementary material for: Palaeoecology of a billion‐year‐old non‐marine cyanobacterium from the Torridon Group and Nonesuch Formation
Source: Palaeontology. 2015 Nov 9;59(1):89–108. doi: 10.1111/pala.12212 (PMC4995629; doi:10.1111/pala.12212)
Supplement: Supplementary file 1 — Fig. S1. Three populations of E. lacustrina with their corresponding size data. [file PALA-59-89-s001.pdf]

SUPPORTING INFORMATION

PALAEOECOLOGY OF A BILLION YEAR OLD NON-MARINE CYANOBACTERIUM FROM THE TORRIDON GROUP AND NONESUCH FORMATION

by PAUL K. STROTHER *and* CHARLES H. WELLMAN

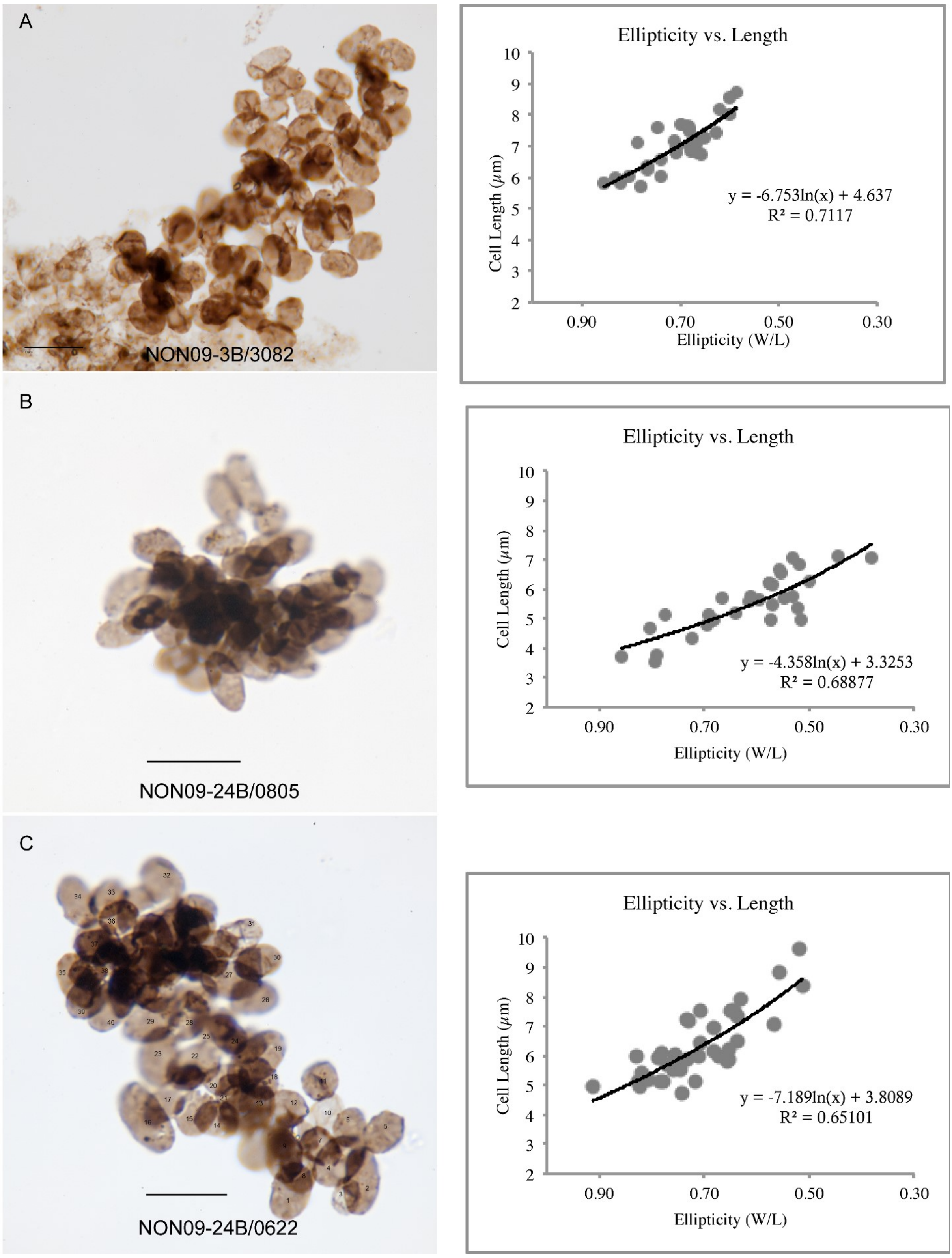

FIG S1. Three populations of *E. lacustrina* with their corresponding size data. A, population from sample NON09-24B with corresponding plot of length vs ellipticity (length/width). B, another population from sample NON09-24B with corresponding plot of length vs ellipticity (length/width). C, population from sample NON09-3B with corresponding plot of length vs ellipticity (length/width).
