# Supplementary material for: Palaeoecology of a billion‐year‐old non‐marine cyanobacterium from the Torridon Group and Nonesuch Formation
Source: Palaeontology. 2015 Nov 9;59(1):89–108. doi: 10.1111/pala.12212 (PMC4995629; doi:10.1111/pala.12212)
Supplement: Supplementary file 2 — Fig. S2. Images from thin section of a phosphatic nodule cut parallel to bedding. [file PALA-59-89-s002.pdf]

SUPPORTING INFORMATION

PALAEOECOLOGY OF A BILLION YEAR OLD NON-MARINE CYANOBACTERIUM FROM THE TORRIDON GROUP AND NONESUCH FORMATION

by PAUL K. STROTHER *and* CHARLES H. WELLMAN

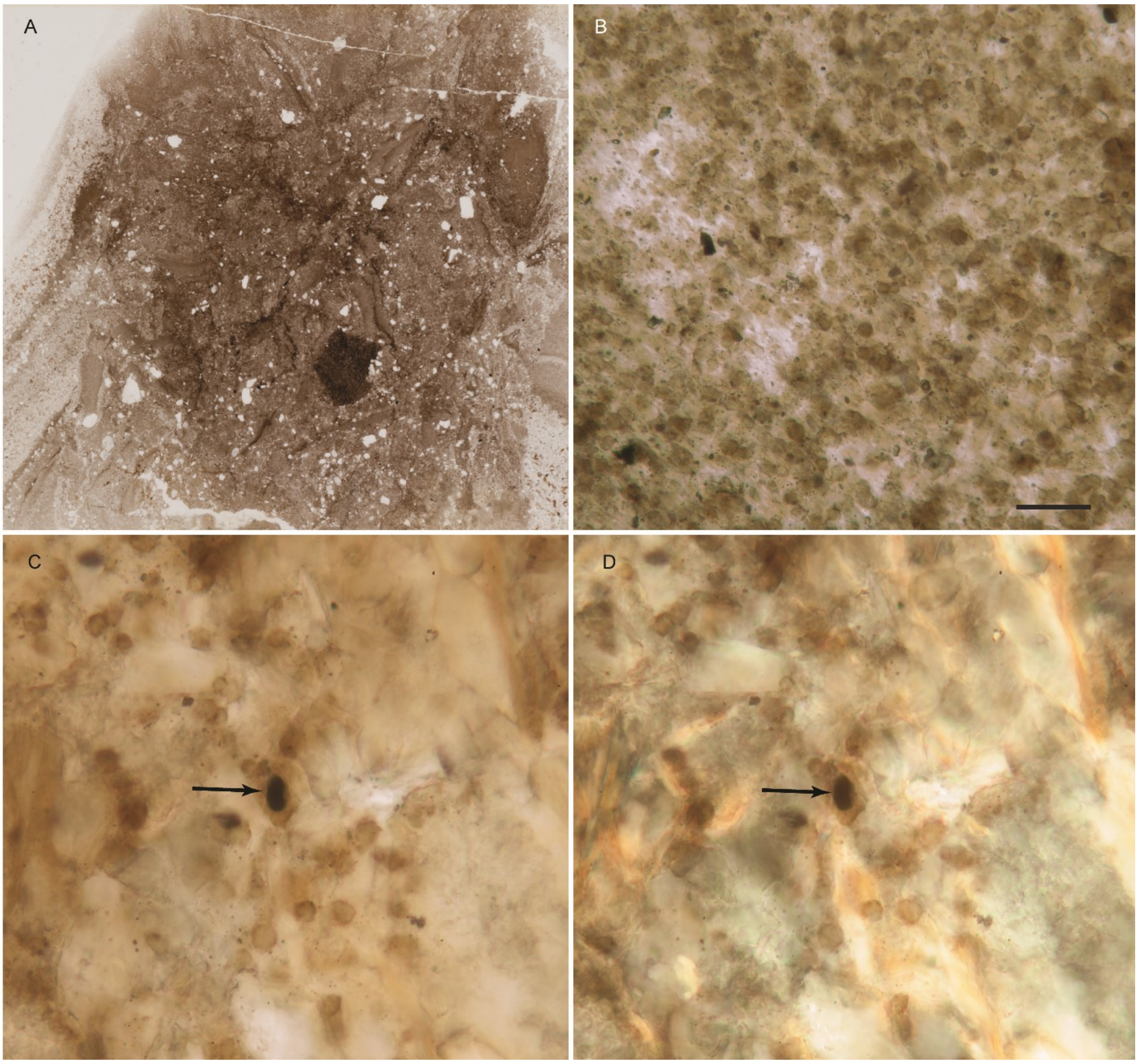

FIG S2. Images from thin-section of a phosphatic nodule (sample number CW1C) cut parallel to bedding, showing the correspondence between distributed cells of *E. lacustrina* and granular phosphate (francolite). A, bedding parallel thin section of a phosphatic nodule, sample number CW1C, which is the source of the section in A; field of view *c.* 2.5 cm. B, region of granular phosphate with associated organic matter. C, a single specimen of *E. lacustrina* (arrow) embedded in a matrix of clay and granular francolite; plane polarized light. The size and distribution of granular phosphate matches the scale of microfossil distributions in well-preserved portions of the nodule (compare to A). D, same as in C, but photographed under crossed nicols. Scale bars represent 25 µm (B); 10 µm (C–D).
